# Hop to It! A Systematic Review and Longitudinal Meta-analysis of Hop Performance After ACL Reconstruction

**DOI:** 10.1007/s40279-024-02121-1

**Published:** 2024-10-16

**Authors:** Michael A. Girdwood, Kay M. Crossley, Ebonie K. Rio, Brooke E. Patterson, Melissa J. Haberfield, Jamon L. Couch, Benjamin F. Mentiplay, Michael Hedger, Adam G. Culvenor

**Affiliations:** 1 La Trobe Sport and Exercise Medicine Research Centre, School of Allied Health, Human Services and Sport, La Trobe University, Bundoora, VIC 3086 Australia; 2 The Australian Ballet, Victoria, Australia; 3 The Victorian Institute of Sport, Victoria, Australia; 4 Arthritis Research Canada, Vancouver, BC Canada; 5 Sport, Performance, and Nutrition Research Group, School of Allied Health, Human Services and Sport, La Trobe University, Melbourne, Australia

## Abstract

**Background:**

Hop testing is widely used by clinicians to monitor rehabilitation and decide when to return to sport following anterior cruciate ligament reconstruction (ACLR); however, the trajectory of long-term hop performance has not been summarised.

**Objective:**

To investigate hop performance change over time after ACLR.

**Design:**

Systematic review with longitudinal meta-analysis.

**Data Sources:**

MEDLINE, EMBASE, CINAHL, Scopus, Cochrane CENTRAL and SPORTDiscus to 28 February 2023.

**Eligibility Criteria:**

Studies with ≥ 50 participants following primary ACLR, with mean participant age of 18–40 years, reporting a quantitative measure of hop performance (e.g. single forward hop distance). Results had to be reported for the ACLR limb and compared with (1) the contralateral limb (within person) and/or (2) an uninjured control limb (between person).

**Results:**

We included 136 studies of 23,360 participants. Performance was similar across different hop tests, with steep initial improvements in within-person symmetry, tailing off after 18–24 months. ACLR limb hop performance was 5–10% lower compared with the contralateral limb at 1 year post-surgery, with largest deficits observed for vertical hop [87.0% contralateral limb (95% CI 85.3–88.8) compared with single forward hop 93.8% (95% CI 92.8–94.9)]. By 3–5 years, results were similar between ACLR and contralateral limbs. There were limited data for between-person comparisons (*n* = 17 studies). Exploratory analyses showed deficits in all forward hopping tests to be very strongly correlated with each other [e.g. single forward and triple hop rho = 0.96 (95% CI 0.90–0.99)], though there was discordance in the relationship between single forward hop and vertical hop performance [rho = 0.27 (95% CI − 0.53 to 0.79)].

**Conclusions:**

Hop performance is comparable to the uninjured limb by 3–5 years post-ACLR, with the greatest deficits in within-person symmetry present in vertical and side hop tests. Assessment of hopping in multiple planes and comparison with uninjured controls, may provide the most complete evaluation of functional performance.

**Supplementary Information:**

The online version contains supplementary material available at 10.1007/s40279-024-02121-1.

## Key Points

**Table:** 

ACLR limb hop performance was 5–10% lower compared with the contralateral limb at 1 year post-ACLR, improving slightly after this time.
There were limited data comparing to uninjured controls, showing deficits of 10–20% across different hop tests.
Results from forward hopping tests were very strongly correlated, but there was a discordant relationship between forward and vertical and side hop tests.

## Introduction

Following anterior cruciate ligament reconstruction (ACLR), objective measures of functional performance underpin monitoring of rehabilitation and assessing readiness to return to sport. The most common method to assess functional performance after ACLR is testing the ability to hop maximally (e.g. hop for distance, hop for height). Hop testing is advocated by clinical practice guidelines and consensus statements as a reliable measure of objective physical function after knee injury [1–3], and is widely used by clinicians given the easy administration and interpretability [4]. Hop test results help guide clinicians’ decision making and confidence in rehabilitation progression or return to sport. For example, better performance on tests such as a single forward hop and triple crossover hop are associated with higher likelihood of return to sport and better self-reported symptoms after ACLR, although the association between hop performance and re-injury risk is inconsistent [4, 5]. Understanding the longitudinal profile of hop performance provides a more thorough understanding of the patient journey than a single timepoint measurement. Surprisingly, despite their widespread use and clinical utility, the trajectory of hop performance after ACLR is not well understood, especially for the medium to long term (i.e. > 1 year post-surgery). Measuring long-term changes in physical capacity could help to understand how the burden of future knee symptoms and osteoarthritis develops. A comprehensive summary of hop performance trajectory after ACLR is needed.

The only systematic review of functional tests (including hopping) after ACLR was conducted almost 10 years ago without meta-analysis, and excluded studies of participants more than 2 years post-surgery [6]. Hop tests such as single forward, triple forward, triple crossover and 6 m timed have been used by clinicians for decades; however, other tests, such as the vertical and side hop, have been recently suggested to highlight potential deficits in knee function, even when symmetry has been achieved in forward hop tests. An updated synthesis is important for clinicians and patients, for benchmarking rehabilitation, providing education on prognosis and expectations, and highlighting research gaps. Concerns have also been raised about using the contralateral limb as a comparator for ACLR limb function (from overestimation of symmetry due to contralateral limb weakening [7]). Others have shown potential gradual improvement in contralateral limb function as a result of rehabilitation [8]. To combat these concerns, comparison with normative data from uninjured controls may provide important context for functional performance findings. Therefore, the aim of this systematic review was to evaluate hop performance changes over time after ACLR using both within-person (compared with the contralateral uninjured limb) and between-person comparisons (compared with uninjured participants). To understand hop performance trajectory, we aimed to use multi-level meta-analyses to leverage information from multiple timepoints, resulting in more efficient and robust estimates of outcomes [9]. A secondary aim was to investigate relationships between performance on different hop tests.

## Methods

This systematic review adhered to Preferred Reporting Items for Systematic Reviews and Meta-Analysis (PRISMA) guidelines [10] and was prospectively registered (PROSPERO: CRD42020216793) alongside other systematic reviews examining muscle strength after ACL injury [11]. There were two protocol deviations: (1) the effect size used in analyses was changed to the ratio of means (RoM) to allow for inclusion of studies reporting limb symmetry index (LSI) as the within-person effect size and (2) additional inclusion criteria were added (publication date from 2010 onwards and total participants *n* ≥ 50) for feasibility purposes given the number of papers captured (> 1100 studies initially included) and to ensure results are drawn from the most contemporary evidence available. All protocol changes were made prior to conducting data analysis.

### Eligibility Criteria

We included peer-reviewed original data studies of primary ACL rupture (with at least 50 participants), who underwent surgical reconstruction and were aged 18–40 years. If a study did not report any revision cases or prior injuries, but did not explicitly state primary injury it was included. No other restrictions were placed on concomitant injury, rehabilitation participation, surgical procedures or study design.

Studies had to report a quantitative measure of hop performance (e.g. distance or count), as a measure of unilateral function. Outcomes relating to quality or biomechanics of movement were excluded. Hop performance had to be reported for the ACLR limb and either (1) the contralateral limb (within-person comparison) or (2) an uninjured control limb (between-person comparison). For within-person comparisons, we included studies that reported values for both limbs or an LSI. We excluded studies that only reported outcomes unilaterally (without comparison), reported only a mean difference or those that did not specify the ACLR limb (e.g. reported only left/right).

### Search Strategy

A search strategy was employed to capture all outcomes within the broader group of systematic reviews. Terms were piloted using key literature, focusing on two elements: population (ACLR) and outcome (muscle strength or functional performance). MEDLINE, EMBASE, CINAHL, Scopus, Cochrane CENTRAL and SPORTDiscus were searched from inception to 28 February 2023 using free text and MeSH terms tailored to each database (Supplement 1). Where possible searches were limited to humans, but not restricted based on language.

### Study Selection

Two authors (MAG and either MJH or BEP) screened title/abstracts against eligibility criteria before assessing full texts for inclusion, resolving any discrepancies with discussion. A third reviewer was consulted for any disagreements as described previously [12].

### Data Extraction

Data were extracted by one author (MAG) using a customised piloted extraction form with data validation where possible and checked by another author (JLC/MJH). Information on study design, country of study, demographics for each group (e.g. age, sample size, number of women, body mass index, graft type), hop test parameters, timepoint of data collection (post-surgery) and outcome data (reported as ACLR limb, contralateral limb, uninjured control limb or LSI). All available timepoints were extracted from longitudinal studies. Graphical data extraction was used where studies presented findings exclusively in plots (Plot Digitizer, v2.6.8, http://plotdigitizer.sourceforge.net/). Non-English studies were translated using DeepL (http://www.deepl.com) [13].

### Risk of Bias Assessment

Risk of bias was assessed independently by two reviewers (MAG and either MJH or BEP) using domains from Cochrane Collaboration guidelines (Supplement 2): random sequence generation, allocation concealment, blinding of therapist and patient, blinding of assessor, outcome measurement, bias in selection of participants, attrition and statistical analysis [14]. We only assessed relevant domains dependent on study design (e.g. bias related to randomisation or group allocation was not assessed in non-randomised studies). Grading was made as high, low or unclear, and disagreements were discussed until consensus, consulting a third reviewer (AGC) if required. We took a pragmatic approach to assessing certainty of evidence and did not formally perform Grade of Recommendations Assessments, Development and Evaluation (GRADE) assessment. Most studies in this review were observational and many with some risk of bias concerns, which would result in automatic downgrades to very low certainty evidence. Acknowledging the studies’ limitations, we opted against performing GRADE assessment, as it was unlikely to add a meaningful interpretation of the evidence synthesis for our research question.

### Data Analysis

To measure change in hop performance over time after ACLR, we conducted several meta-analyses using RStudio [15]. The RoM was used as the effect size for all analyses [16], which expresses the effect as the mean of one group divided by the mean of the comparator (e.g. in our case ACLR limb compared with contralateral limb or uninjured control limb). Calculation of variance is previously published [16, 17], including adjusting for correlation of within-person comparisons (i.e. ACLR limb versus contralateral). As between-limb correlation is usually not reported, we estimated this based on our own data as *r* = 0.85 [18–20]. Using the RoM simplifies clinical interpretation compared to metrics such as standardised mean difference (e.g. a RoM of 0.8 indicates the ACL limb is 0.8 × the performance of the comparator, or a 20% deficit) [16]. It is widely used in ecological research and allows for inclusion of studies which report only LSIs (a mean of ratios, which we approximated to the RoM using appropriate formulae (Supplement 3)). Findings not reported as mean and standard deviation were transformed as recommended [14, 21]. Missing standard deviation data were imputed using the ‘simputation’ package using predictive mean matching [22]. Multiple studies of the same cohort (e.g. across different papers/author groups) were tracked and classified by a ‘cohort’ variable, to maximise longitudinal analysis, but were checked for any data duplication (e.g. baseline hopping data presented across different papers). Multiple groups per cohort were combined at each timepoint to result in one effect per timepoint per study (Supplement 3).

Data were pooled based on the type of hop test (e.g. single forward hop) and the type of comparison: (1) within person (compared with contralateral limb) or (2) between person (compared with uninjured control group), selecting the dominant limb of control groups if possible. Timepoints from < 2 months post-ACLR were not included in meta-analyses given the post-surgical limitations and restrictions often seen at this time.

We conducted longitudinal mixed-effects meta-analyses using a restricted maximum likelihood estimator, similar to previous methods [23, 24], using the ‘metafor’ package [25]. We used a correlated hierarchical effect model [26] to account for cohorts contributing multiple correlated effects over time. We piloted different working model structures, with final models including random effects for timepoints nested within cohorts using a continuous auto-regressive (CAR) sample correlation structure. Addition of further random effects was trialled but led to overly complex models with indistinguishable random effects. We then added a moderator variable for timepoint (fixed effect), to evaluate the change in hop performance over time. We trialled fitting timepoint as a linear, log-linear, polynomial, 3-knot spline and 4-knot spline using model heuristics [Akaike Information Criterion (AIC), Bayesian Information Criterion (BIC)], as well as visual inspection of model fit to determine the most suitable approach. Splines were fit with the ‘rms’ package [27], with knot locations specified as recommended [28]. Robust variance estimation was used in final models (using the ‘clubSandwich’ package [29]). Details of final models are available in Supplementary Table 6.1. Results are presented as the predicted model estimates and 95% confidence intervals, along with prediction intervals as a measure of heterogeneity (estimate of where future observations could fall) [30].

There was insufficient data to conduct longitudinal analysis for most between-person comparisons (except for single forward hop), so univariate meta-analyses were conducted with a restricted maximum likelihood estimator, pooling separately for each hop test.

As an exploratory secondary analysis, we investigated the relationship between performance on different hop tests where studies reported results for more than one hop test, using a bivariate model [31]. This allowed us to understand the estimated correlation between two hop tests and to regress the estimated true effects to understand the slope relationship. On this analysis, each study contributes two correlated effects (e.g. single forward hop and triple forward hop from the same sample). We used only one timepoint from each study, taking the closest to 12 months post-ACLR from multi-timepoint studies. A correlated hierarchical effect model was fit with random effects for effect type (e.g. single forward hop and triple forward hop), nested within each cohort, as well as a moderator variable (fixed effect) for effect type. A second regression model was then fit with the random effects and estimated variance–covariance matrix from the multivariate model, to provide an estimate of the slope relationship between the two hop tests [31, 32]. We report the results as the estimated correlation between the true hop test effect sizes (rho, < 0.4 = weak, 0.41–0.60 = moderate, 0.61–0.80 = strong, > 0.80 = very strong) as well as the estimated slope relationship between hop tests. We performed this analysis for the most common combinations of tests reported (i.e. ≥ 10 studies reporting the combination): (1) single forward hop and triple forward hop, (2) triple forward hop and triple crossover hop, (3) single forward hop and triple crossover hop, (4) single forward hop and 6 m timed hop, (5) single forward hop and vertical hop, and (6) single forward hop and side hop.

We assessed publication bias (for comparisons with *n* ≥ 10), using a modified Egger regression test with robust variance estimation, accounting for potential dependence of effect sizes [33]. We conducted a sensitivity analysis to investigate whether graft type affected hop performance. We compared the two most commonly reported graft types [hamstring, bone–patellar tendon–bone (BPTB), if *n* ≥ 10 studies per group], by adding a graft grouping term as a moderator variable to our final models with an interaction term (removed if non-significant). Results are presented with plots of the trajectory for each graft group, as well as pairwise comparisons constructed with the ‘emmeans’ package.

Any data that could not be summarised quantitatively were synthesised narratively. Scripts and all data used are publicly available: https://github.com/mgirdwood7/hop_sr.

## Results

**Fig. 1:**
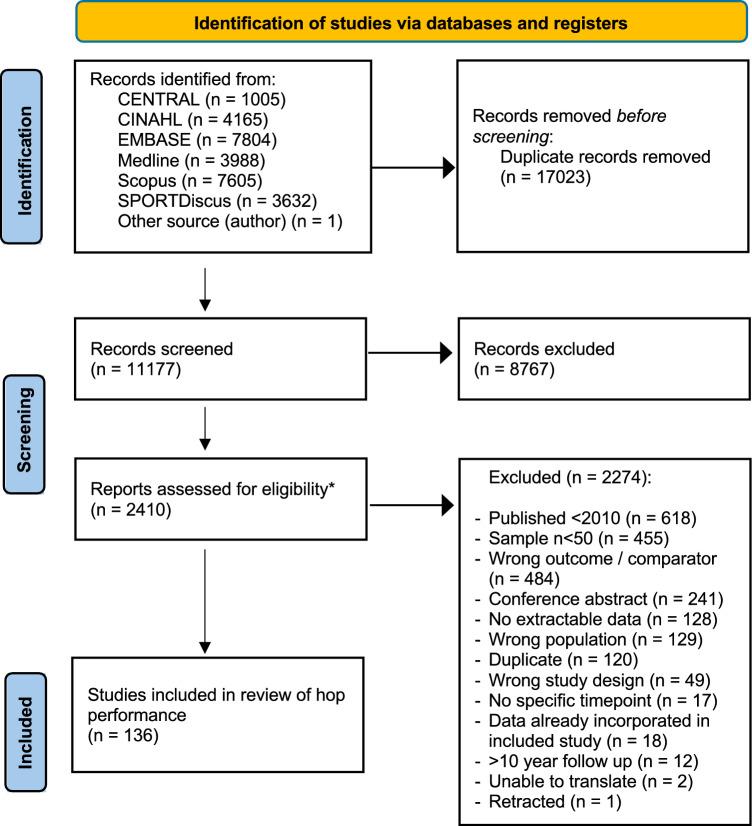
PRISMA flow chart of included studies

We included 136 studies of 23,360 participants (21,876 ACLR, 1484 uninjured control) from 26 countries [8407 (36%) women] (PRISMA flow chart, Fig. 1). Most studies (*n* = 132) compared hop performance within person; only 17 (13%) compared between person. Median total ACLR sample size was 83 [interquartile range (IQR) 64–144]. The most common timepoints post-ACLR for hop assessment were 6 months (*n* = 36), 1 year (*n* = 31) and 2 years (n = 15), with 40 cohorts reporting hop results across multiple timepoints (median 2, maximum 6),

Of the 21,876 included participants with ACLR, 38% (8324) received flexor grafts (any type of hamstring graft), 8% (1837) extensor grafts (bone–patellar tendon–bone or quadriceps tendon), graft type was mixed or unclear for 46% (9998) (participant groups included a mixture of graft types or graft not specified) and 8% (1717) other graft groups (e.g. tibialis anterior). Mean age ranged from 18 to 38 years (median 27, IQR 22–29 years). Mean BMI was reported by 59 studies, ranging from 21.9 to 28.0 kg/m^2^ (median 24.3, IQR 23.6–25.0 kg/m^2^).

Hop test methodology was often poorly reported, with 115 studies (85%) not providing details on hand placement and 103 studies (76%) not detailing landing requirements. Of those that did provide detail on upper limb placement (*n* = 21 studies), there was limited consistency in hand placement (*n* = 14 with hands on hips or behind back, *n* = 7 arms free). Detail provided on landing instructions (*n* = 33 studies) generally all focused on a stable landing constraining subsequent movement, though there were subtle differences between studies (e.g. stable landing for 1, 2 or 3 s). Eighty-two studies (60%) cited other papers for their methodology (32 unique papers cited).

### Risk of Bias

Most studies were at high or unclear (*n* = 121, 90%) risk of assessor blinding (Supplement 5.1, 5.2). Twenty-three studies (17%) were at high risk of bias for outcome measurement—i.e. did not provide replicable detail on the methods of hop testing. Selection bias was high or unclear in 37 (27%) studies and in 71% of between-person studies. Removal of 11 (8%) studies in a sensitivity analysis, rated high risk of bias for assessor blinding, outcome measurement and selection, did not alter pooled estimates (Supplement 11).

### Within-Person Differences

**Fig. 2:**
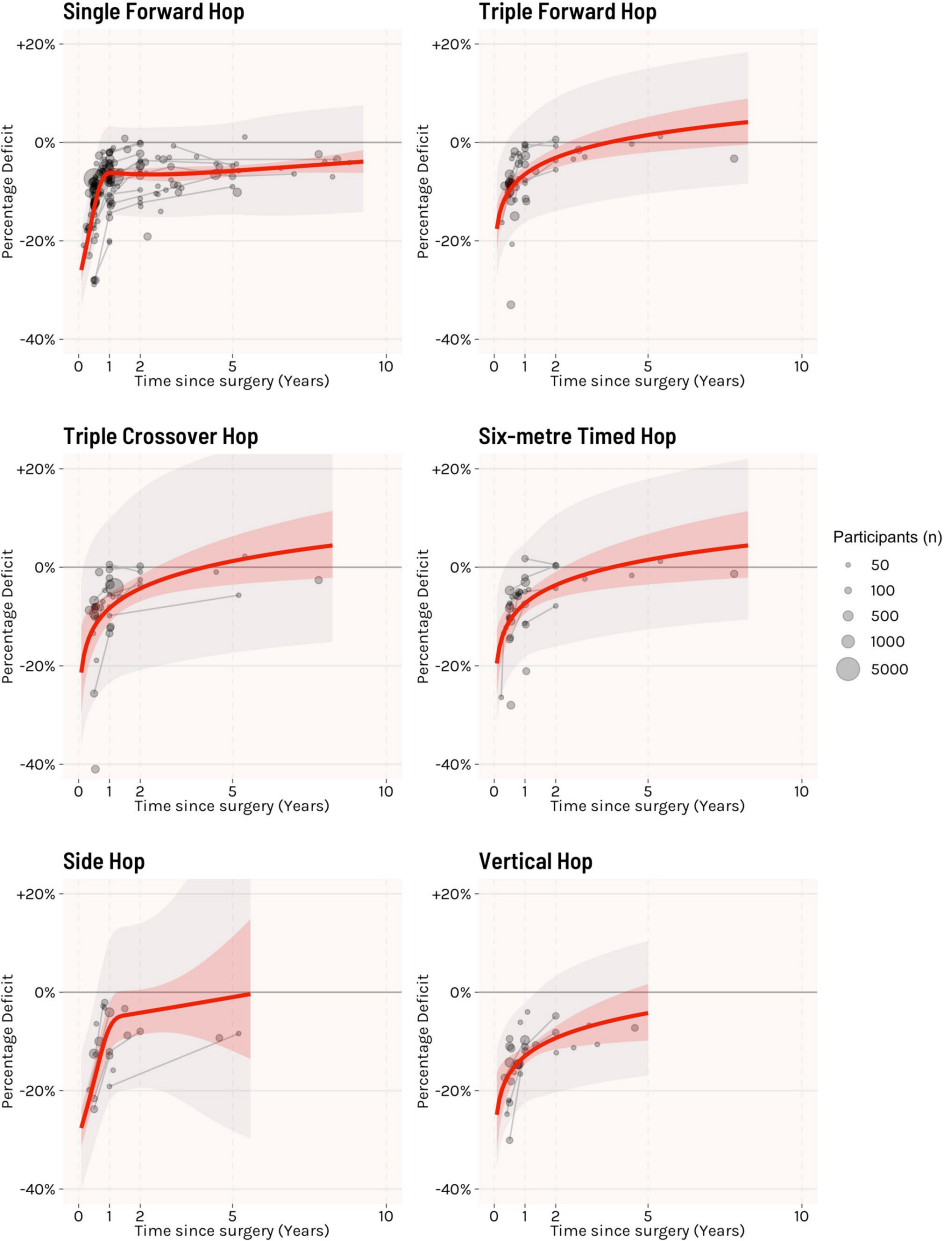
Meta-analysis of within-person comparisons for different hop tests. The red line and shaded region represent the estimated fit (ratio of means, expressed as percentage deficit) and 95% confidence interval, respectively, with grey shading the prediction interval. Grey dots represent individual cohorts with lines showing linked timepoints across cohorts. *n* = total sample size, *k* = number of individual effects

Performance on all tests followed similar trajectories, with initial improvements plateauing after 18–24 months (Fig. 2, Table 1). Performance of the ACLR limb was lower than the contralateral limb for triple forward, triple crossover and 6 m timed hop tests up to approximately 2.5 years, with slightly slower recovery for vertical hop (estimated 4.3 years) (Table 1). Single forward hop performance was lower for all timepoints reported (up to 8.8 years). Estimated mean hop performance across all tests at 1 year ranged from 87.0% to 93.8% of the contralateral limb, with the lowest pooled estimate for vertical hop. At 2 years, there was only slight additional improvement, with mean estimates approximately 95% of the contralateral limb, and vertical hop was again the lowest. By 5 years, most tests did not show significant differences within person, with only single forward hop still with deficits. We found evidence of potential publication bias for single forward hop, triple forward hop and 6 m timed hop, but not for other hop tests (Supplement 8). A summary of other less commonly used hop tests is provided in Supplement 9.

**Table 1:** Summary of longitudinal meta-analyses of within-person comparisons of hop performance, separated by each hop test

Hop test	*n* (total sample)	Total studies [total effects (*k*)]	1-year estimate	2-year estimate	5-year estimate	Estimated timepoint of no difference (years)	Last data point (years)
Single forward hop	19,239	120 (158)	93.8 (92.8–94.9)	93.5 (92.3–94.7)	94.2 (93.1–95.3)	NA	8.8
Triple forward hop	4993	40 (51)	93.5 (92–95.1)	96.9 (94.9–98.9)	101.5 (98–105.2)	2.3	7.8
Triple crossover hop	5596	29 (39)	91.7 (88.7–94.7)	95.7 (93.1–98.4)	101.2 (96.4–106.3)	2.5	7.8
Six-metre timed hop	2710	25 (38)	92.7 (90.6–94.8)	96.4 (93.7–99.1)	101.5 (96.5–106.8)	2.3	7.8
Side hop	1613	14 (19)	92.8 (87.9–98)	95.8 (91.3–100.5)	99 (88.1–111.3)	1.3	5.2
Vertical hop	2850	23 (28)	87 (85.3–88.8)	90.7 (88.4–93.1)	95.8 (90.2–101.7)	4.3	4.6

Estimates are a ratio of means (RoM) and 95% confidence interval, interpreted as the percentage of the contralateral limb

Estimated timepoint of no difference represents the timepoint where 95% confidence interval crosses 100% limb symmetry (i.e. no difference between limbs)

*NA* not applicable

### Between-Person Differences

Longitudinal meta-analysis was only possible for single forward hop (15 studies, 19 effects; Table 2, Supplement 7.1), showing lower performance compared with uninjured controls up to at least 2.8 years. Estimated single forward hop performance at 1 year was 86.8% [95% confidence interval (CI) 82.4 to 91.4] and 90.5% (95% CI 85.1 to 96.3) at 2 years.

**Table 2:** Summary of meta-analyses of between-person comparisons of hop performance, separated by each hop test. Estimates are a ratio of means (RoM) and 95% confidence interval, interpreted as the percentage of the contralateral limb.

Hop test	*n* (total sample)	Total studies	Pooled estimate			Range of timepoints pooled (years)
Results from longitudinal meta-analysis			1-year estimate	2-year estimate	5-year estimate	
Single forward hop	1010	15	86.8 (82.4–91.4)	90.5 (85.1–96.3)	95.7 (84.9–107.8)	0.5–5.2

Hop test	*n* (total sample)	Total studies	Overall estimate	Range of timepoints pooled (years)
Results from univariate meta-analysis				
Triple forward hop	296	4	87.7 (94.9–90.5)	0.5–0.7
Triple crossover hop	159	3	86.5 (81.6–91.8)	0.5–1
Six-metre timed hop	100	2	80.5 (66.8–97.0)	0.5–0.7
Side hop	350	4	85.1 (76.3–94.9)	1.5–5.2
Vertical hop	288	4	89.4 (79.6–100.4)	0.5–4.5

Longitudinal analysis was only possible for single forward hop

For all other tests, only univariate meta-analysis was possible (Table 2, Supplementary Figs. 7.2–7.6). Pooled mean estimates of hop performance ranged from 80.5% to 89.4% of uninjured control limbs, predominantly driven by studies in the first year post-surgery.

### Relationship Between Different Hop Tests

**Fig. 3:**
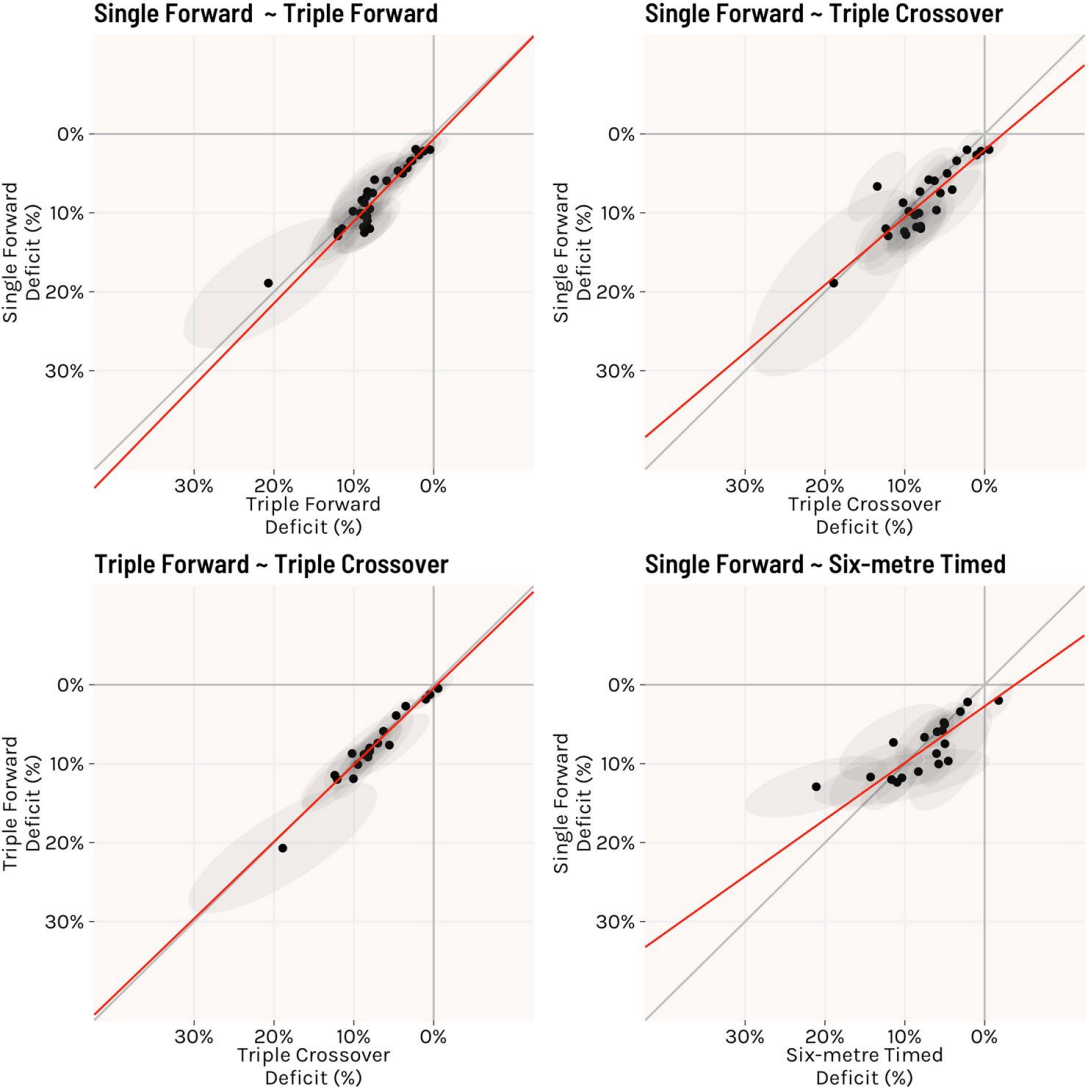
Plots showing relationship between estimated true deficits of different forward hop tests. The black dots display point estimates from individual studies, with grey shading representing the 95% confidence ellipse. The red line represents the slope relationship between hop tests. Grey diagonal line = perfect 1:1 relationship

Exploratory analysis from studies reporting results of multiple hop tests showed that performances on forward (horizontal) hop tests were highly correlated (Fig. 3), with a very strong (close to perfect) relationship in performance deficits between single forward hop and triple hop (rho = 0.96, 95% CI 0.90–0.99; slope = 1.04, 95% CI 0.89–1.19) and triple forward hop and triple crossover hop (rho = 0.98. 95% CI 0.94–0.99; slope = 0.97, 95% CI 0.85–1.09). There was also a very strong relationship between single forward hop and triple crossover hop (rho = 0.86, 95% CI 0.67–0.95; slope = 0.86, 95% CI 0.61–1.10), single forward hop and 6 m timed hop (rho = 0.87, 95% CI 0.63–0.96; slope = 0.72, 95% CI 0.45–0.98) and 6 m timed hop and triple hop (rho = 0.95, 95% CI 0.85–0.98; slope = 0.85, 95% CI 0.69–1.01). There was a strong relationship between 6 m timed hop and triple crossover hop (rho = 0.79, 95% CI 0.47–0.93; slope = 0.83, 95% CI 0.47–1.19).

**Fig. 4:**
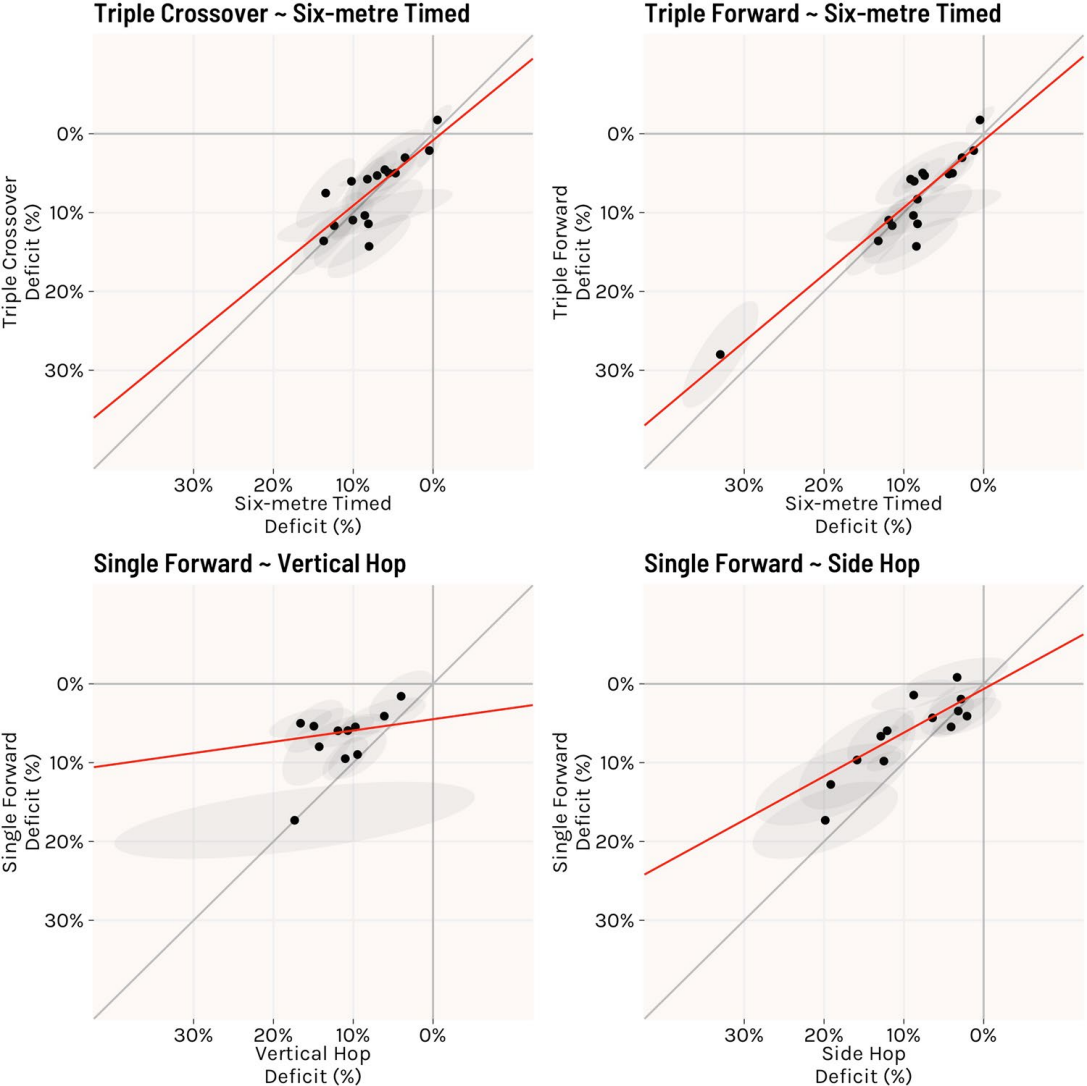
Plots showing the relationship between estimated true deficits of single forward hop and vertical hop (left) and side hop (right). The black dots display point estimates from individual studies, with grey shading representing the 95% confidence ellipse. Red line represents the slope relationship between hop tests. Grey diagonal line = perfect 1:1 relationship

The relationship between single forward hop and vertical hop test was discordant (Fig. 4), with major variability present (rho = 0.27, 95% CI − 0.53 to 0.79; slope = 0.14, 95% CI − 0.27 to 0.56), suggesting vertical hop deficits within person were not related to single forward hop deficits. Single forward hop and side hop also showed a moderately diverging relationship, with deficits in side hop estimated to be 1.8-fold larger than side hop (rho = 0.81, 95% CI 0.41–0.95; slope = 0.55, 95% CI 0.28–0.83).

### Differences in Hop Performance Between Grafts

Comparison of hop performance between hamstring and BPTB could only be performed for single forward hop measured within person. There was a very small difference between grafts (Supplementary Fig. 10.1), with 1.01-fold greater hop performance for hamstring grafts compared with BPTB (95% CI 1.00–1.03). There were insufficient data available for graft comparisons for any other hop test.

## Discussion

We undertook the most extensive review of hop performance after ACLR to date, including data from 136 studies and over 23,000 participants, focusing on six common hop tests. Despite the large number of included studies, most evidence was low quality, being observational in nature with at least some risk of bias concerns. Performance of the ACLR limb was lower than the contralateral limb by approximately 5–10% at 1 year post-surgery and was within 6% at 5 years post-ACLR across all hopping tests. Data comparing with uninjured controls were limited, with results showing slightly greater deficits (10–20%), mostly from timepoints in the first 2 years post-surgery. Prediction intervals were wide for most analyses, highlighting the clinical variability seen in post-ACLR populations and the potential impact of subtle differences in testing methods often inadequately reported by studies [34]. We found a very strong correlation between forward hop test variations, with more inconsistency between hops of different direction (e.g. forward versus side or vertical hop). This suggests hop tests in different planes may assess different elements of lower limb function, supporting their use for comprehensive functional assessment and guidance of return-to-sport decisions.

Understanding hop performance recovery over time following ACLR is critical as hop performance is a commonly used benchmark by clinicians and researchers as part of return-to-sport testing and rehabilitation monitoring [35]. A hop performance target of 90% compared with the uninjured limb (i.e. LSI) is commonly set as a rehabilitation goal [36]. Achieving this milestone is associated with five-fold reduced re-injury risk upon return to sport and halves the odds of knee osteoarthritis (OA) development [5]. LSI milestones are also useful for motivating patients to complete late stage rehabilitation and reach their goals [36]. The single forward hop continues to be the most commonly used test (almost 90% of studies in this review), with persistent deficits seen at all timepoints included, though potentially less clinically meaningful at longer-term follow-ups (> 5 years), given measurement error of approximately 5% [37]. The limited data in this review comparing with uninjured controls (predominantly from the first 12 months post-ACLR) suggest that despite potentially ‘normal’ symmetry (i.e. > 90% contralateral limb), deficits in hop performance may still exist when compared to uninjured people (normative data). It is unclear though how relevant longer term within-person deficits of 5% or less are to ongoing knee health or patient goals, especially if someone has stopped playing sport or modified their activities.

Evaluating only forward hop tests is likely to insufficiently evaluate lower-limb functional capacity. Vertical and side hop tests showed the greatest within-person deficits, with vertical hopping in particular remaining approximately 10% lower than the contralateral limb at 2 years post-surgery. Our results add to growing evidence that vertical hopping may highlight movement deficits (biomechanics and performance) differently to other hop tests after knee injury [38], as it has up to three-fold higher knee work contribution than horizontal hopping during the propulsion phase [39, 40]. Our exploratory analysis showed that the relationship between single forward hop and vertical and side hop were discordant (i.e. not fully representative of each other), suggesting they may be testing different constructs. The vertical hop and side hop may challenge knee confidence more than forward hopping [41], which could make it sensitive for guiding return-to-sport decision making. The side hop test is also less commonly utilised (10% of studies in this review), though given the components of dynamic stability, energy storage, endurance and movement confidence [42, 43], it seems valuable for comprehensive functional assessment.

There was a significant evidence gap for between-person comparisons (i.e. uninjured controls), with only 17 studies measuring this (compared with 132 for within-person comparisons), and most only tested the single forward hop. Most data from this review were drawn from LSIs, which may provide imprecise estimates of actual ACLR limb function. The contralateral limb may be a biased comparator as it often exhibits lower performance after ACLR [7, 20], stemming from lower activity levels, and bilateral central nervous system changes [44, 45]. Conversely, other studies have shown improvements in contralateral limb function post-ACLR [8, 46], highlighting the challenge of using it as a comparator. We only had sufficient data to conduct longitudinal meta-analysis for single forward hop, with results possibly pointing to greater deficits than seen within person, consistent with our review on thigh muscle strength [47]. Normative data for hop performance are scarce for those aged > 25 years, especially for non-athletic populations [48]. Even though the ACL is commonly injured playing sport, almost half do not return to sport [49], and benchmarks from athletic controls may not be ideal. Future studies with larger sample sizes comparing ACLR hop performance with well-matched uninjured controls will help to understand the true deficits in hop performance across the lifespan after ACLR and provide normative benchmarks for clinicians during rehabilitation.

For clinicians, hop performance may continue to improve somewhat after the first year of rehabilitation post-ACLR, and holistic monitoring alongside muscle strength, biomechanics, movement confidence and psychological readiness will provide a broad assessment of knee health status following ACLR. State-of-the-art clinical recommendations from a recent consensus statement (OPTIKNEE) emphasise the importance of completing a battery of hop tests in vertical, forward and lateral directions to encompass all possible movement deficits [3]. Our findings endorse this advice, and especially encourage measurement of tests such as the vertical and side hop which might be more sensitive to symmetry deficits in hop performance, in addition to forward hop tests [4]. The triple, triple crossover and 6 m timed hop all followed similar trajectories of recovery and effect sizes were almost perfectly correlated (exploratory analysis). These tests likely measure very similar constructs, as all involve repeated hopping/bounding of a similar distance (e.g. triple hop results are often around 4–5 m) [4], so clinicians may consider choosing one for efficiency. When assessing patients, to ensure the most robust estimate of physical function, we suggest benchmarking against relevant normative data if available (i.e. sport or sex specific) [48], in addition to within-person comparisons (measured by LSI). In future, it may also be useful to explore whether establishing a threshold for optimal hop performance (e.g. relative to leg length or height) can be useful for clinical decision making. It is also important to consider movement quality during hopping where biomechanical deficits may be masked by purely performance based metrics (e.g. distance) [4].

There were several limitations of this review. Though we leveraged data from > 23,000 people and 136 studies to strengthen our findings, this has led to heterogeneity in outcomes that reflects the variety of clinical pathways and presentations after ACLR. The large number of included studies, however, gives us confidence that our estimates are a robust estimation of current practice, encompassing the true mean effect. Another limitation was a significant lack of between-person comparisons for most hop tests. Within-person comparisons potentially underestimate the level of dysfunction [7, 20], which could result in erroneous return-to-sport or prognostic advice. Though case–control studies have their own challenges (e.g. selection bias), more are needed focusing on functional performance to investigate the significance of longer-term changes after ACLR. Subtle differences in hop testing methods (e.g. whether hands were free or held behind the back, testing order/fatigue effects) may also have added to the variability of our findings. Unfortunately, most papers provided insufficient detail to reproduce their testing methods—a common issue with hop testing [34], making it difficult to assess the impact of any testing methods or apply specific selection criteria for this review. More detail in reporting testing methods will enable more robust analysis and interpretation of findings from hop tests (i.e. better ability to discriminate whether the variability in estimates is related to the test or to the exposure).

Rehabilitation and adherence to training are key influences on physical performance recovery after ACLR, and this confounder was not considered in this review. Rehabilitation reporting varied significantly with no standardised approach, and observational studies (78% in this review) often did not focus on this. In the general population, rehabilitation adherence varies widely (e.g. only 55% of people saw a therapist after 3 months post-surgery, and most cease rehabilitation before 12 months post-surgery) [50]. Rehabilitation lasting over 6 months is associated with better physical and subjective outcomes [50], though there appear to be no differences in outcomes between supervised and non-supervised approaches [51]. Further research is needed to determine the degree to which training type, duration and frequency impact on physical function and whether an optimal or minimum dosage exists, as well as whether pre-injury activity levels, sex/gender or rehabilitation protocols impact long-term recovery trajectories.

Our results are generalisable to young adults (age 18–30 years) from Western countries, and results may differ for other populations. The impact of access to care and rehabilitation is beyond the scope of this manuscript, but likely affects recovery of physical performance after ACLR. Only one-third of included participants were women, though this potentially reflects the proportion of ACL injuries in society currently. Sex/gender specific differences in hop performance may exist that should be explored in future research [52]. This review focused on hop performance, but many other functional tests are used by clinicians to monitor physical function and rehabilitation, such as the one-leg rise [53] or star excursion balance tests. Standardising and synthesizing their data may provide additional clinical information not captured in hop tests.

## Conclusions

ACLR limb hop performance is lower by approximately 5–10% compared with the contralateral limb at 1 year post-ACLR, but is similar by 3–5 years post-ACLR. Vertical and side hop may show greater deficits or slower recovery compared with the single forward hop when measured within person. Between-person comparisons were lacking in this review, with more research required to understand whether hop performance is altered compared with population norms. A battery of hop tests encompassing multiple planes (forward, sideways, vertical) may provide a useful benchmark of physical function after ACLR, with further work required to determine the clinical utility of hop performance and its association with outcomes.

## Supplementary Information

Below is the link to the electronic supplementary material.

Supplementary file1 (PDF 2627 KB)

## Declarations

### Availability of data and material

Full dataset and analysis scripts available from https://github.com/mgirdwood7/hop_sr.

### Code availability

Full dataset and analysis scripts available from https://github.com/mgirdwood7/hop_sr.
