# Supplementary material for: Hop to It! A Systematic Review and Longitudinal Meta-analysis of Hop Performance After ACL Reconstruction
Source: Sports Med. 2024 Oct 16;55(1):101–13. doi: 10.1007/s40279-024-02121-1 (PMC11787245; doi:10.1007/s40279-024-02121-1)
Supplement: Supplementary file 1 — Supplementary file1 (PDF 2627 KB) [file 40279_2024_2121_MOESM1_ESM.pdf]

Supplementary File

Table of Contents

Supplement 1: Search Strategy ***3***

Table 1.1: MEDLINE Search 3

Table 1.2: EMBASE Search 4

Table 1.3: CINAHL Search 5

Table 1.4: Scopus Search 6

Table 1.5: Cochrane CENTRAL Search 7

Table 1.6: SPORTDiscus Search 8

Supplement 2: Risk of Bias Assessment ***9***

Table 2.1: Risk of Bias Assessment 9

Supplement 3 - Formulae used in data analysis ***12***

Supplement 4 – Included Study Summary ***13***

Supplement 5 – Risk of Bias Summary ***24***

Figure 5.1: Summary of risk of bias assessment. 24

Figure 5.2: Summary of risk of bias assessment. 25

Supplement 6 – Details of final models presented in results ***26***

Table 6.1: Final model parameters 26

Supplement 7 – Results for between-person comparisons ***27***

Figure 7.1: Meta-analysis of between-person comparisons of single forward hop performance. 27

Figure 7.2: Meta analysis of between person comparisons of triple forward hop performance. ACLR = anterior cruciate ligament reconstruction; RoM = ratio of means, CI = confidence interval. 28

Figure 7.3: Meta analysis of between person comparisons of triple crossover hop performance. ACLR = anterior cruciate ligament reconstruction; RoM = ratio of means, CI = confidence interval. 28

Figure 7.4: Meta analysis of between person comparisons of six metre timed hop performance. ACLR = anterior cruciate ligament reconstruction; RoM = ratio of means, CI = confidence interval. 29

Figure 7.5: Meta analysis of between person comparisons of side hop performance. ACLR = anterior cruciate ligament reconstruction; RoM = ratio of means, CI = confidence interval. 29

Figure 7.6: Meta analysis of between person comparisons of vertical forward hop performance. ACLR = anterior cruciate ligament reconstruction; RoM = ratio of means, CI = confidence interval. 30

Supplement 8 – Publication bias assessment ***31***

Supplement 9 – Results for other hop tests ***32***

Supplement 10 – Sensitivity analysis for graft type ***33***

Figure 10.1: Sensitivity analysis for effect of graft type on single forward hop performance. 33

Supplement 11 – Sensitivity analysis removing high risk of bias studies from within-person comparisons ***34***

Figure 11.1 – Single forward hop sensitivity analysis removing high risk of bias studies. 34

Figure 11.2 – Triple forward hop sensitivity analysis removing high risk of bias studies.. 34

Figure 11.3 – Triple crossover hop sensitivity analysis removing high risk of bias studies. 35

Figure 11.4 – Six metre forward hop sensitivity analysis removing high risk of bias studies. 35

Figure 10.5 – Side hop sensitivity analysis removing high risk of bias studies................. 36

Supplement 12 – References for included studies ***37***

Supplement 1: Search Strategy

Table 1.1: MEDLINE Search

Search	Term	Search Detail - Medline Example
		**Element 1 - Anterior Cruciate Ligament**
1	anterior cruciate ligament	anterior cruciate ligament.mp OR exp *Anterior Cruciate Ligament/ OR *Anterior Cruciate Ligament Reconstruction/ OR *Anteiror Cruciate Ligament Injuries/
2	ACL	ACL.mp
		**Combine all with OR**
		**Element 2 - Strength**
2	Strength	muscle strength.mp OR Muscle Strength/
3		knee adj2 strength.mp
4		quad* strength.mp
5		hamstring strength.mp
6		flex* adj2 strength OR ext* adj2 strength
7		hip adj2 strength
8		calf adj2 strength
9	Torque	Torque/ or torque.mp
10	Isokinetic	isokinetic.mp
11	Dynamometry	dynamomet*.mp
12	Quadriceps	quadriceps muscle.mp or Quadriceps Muscle/
13	Hamstring	hamstring muscle.mp or Hamstring Muscles/
14		**Combine all with OR**
		**Element 3 - Function**
16	Hop	hop.mp
17	Jump	jump.mp
18	Functional test	function* adj2 test.mp
19		function* adj2 scores
20		function* adj2 outcome*
21		function* adj2 measure*
22		**Combine all with OR**
		**Final Search**
24		**Combine - Element 1 AND (Element 2 OR 3)**
25		limit to humans

Table 1.2: EMBASE Search

Search	Term	EMBASE
		**Element 1 - Anterior Cruciate Ligament**
1	anterior cruciate ligament	anterior cruciate ligament.mp OR exp *Anterior Cruciate Ligament/ OR *Anterior Cruciate Ligament Reconstruction/ OR *Anteiror Cruciate Ligament Injuries/ OR Anterior Cruciate Ligament Rupture
2	ACL	ACL.mp
		**Combine all with OR**
		**Element 2 - Strength**
2		muscle strength.mp OR Muscle Strength/
3		knee adj2 strength.mp
4	Strength	quad* strength.mp
5		hamstring strength.mp
6		flex* adj2 strength OR ext* adj2 strength
7		hip adj2 strength
8		calf adj2 strength
9	Torque	Torque/ or torque.mp
10	Isokinetic	isokinetic.mp
11	Dynamometry	dynamomet*.mp
12	Quadriceps	quadriceps muscle.mp or Quadriceps Femoris Muscle/
13	Hamstring	hamstring muscle.mp or Hamstring Muscles/
14		**Combine all with OR**
		**Element 3 - Function**
16	Hop	hop.mp
17	Jump	jump.mp
18		function* adj2 test.mp
19	Functional test	function* adj2 score*
20		function* adj2 outcome*
21		function* adj2 measure*
22		**Combine all with OR**
		**Final Search**
24		**Combine - Element 1 AND (Element 2 OR 3)**
25		limit to humans

Table 1.3: CINAHL Search

Search	Term	CINAHL
**Element 1 - Anterior Cruciate Ligament**		
1	anterior cruciate ligament	(MH "Anterior Cruciate Ligament") OR "anterior cruciate ligament" OR (MH "Anterior Cruciate Ligament Reconstruction")
2	ACL	ACL
		**Combine all with OR**
**Element 2 - Strength**		
2	Strength	(MH "Muscle Strength") OR "muscle strength"
3		knee N2 strength
4		quad* strength
5		hamstring strength
6		flex* N2 strength OR extens* N2 strength
7		hip N2 strength
8		calf N2 strength
9	Torque	MH ("Torque") or torque
10	Isokinetic	isokinetic
11	Dynamometry	dyn#momet*
12	Quadriceps	quadriceps muscle
13	Hamstring	hamstring muscle
14		**Combine all with OR**
**Element 3 - Function**		
16	Hop	hop
17	Jump	jump
18	Functional test	function* N2 test
19		function* N2 scores
20		function* N2 outcome*
21		function* N2 measure*
22		**Combine all with OR**
**Final Search**		
24		**Combine - Element 1 AND (Element 2 OR 3)**

Table 1.4: Scopus Search

Search	Term	Scopus
		**Element 1 - Anterior Cruciate Ligament**
1	anterior cruciate ligament	"anterior cruciate ligament"
2	ACL	ACL
		**Combine all with OR**
		**Element 2 - Strength**
2	Strength	"muscle strength"
3		knee W/2 strength
4		"quad* strength"
5		"hamstring strength"
6		flex* W/2 strength OR ext* W/2 strength
7		hip adj2 strength
8		calf adj2 strength
9	Torque	torque
10	Isokinetic	isokinetic
11	Dynamometry	dyn?mometry
12	Quadriceps	"quadriceps muscle"
13	Hamstring	"hamstring muscle"
14		**Combine all with OR**
		**Element 3 - Function**
16	Hop	hop
17	Jump	jump
18	Functional test	function* W/2 test
19		function* W/2 scores
20		function* W/2 outcome*
21		function* W/2 measure*
22		**Combine all with OR**
		**Final Search**
24		**Combine - Element 1 AND (Element 2 OR 3)**

Table 1.5: Cochrane CENTRAL Search

Search	Term	CENTRAL
**Element 1 - Anterior Cruciate Ligament**		
1	anterior cruciate ligament	MeSH [Anterior Cruciate Ligament]
2	ACL	ACL
		**Combine all with OR**
**Element 2 - Strength**		
2	Strength	MeSH [Muscle Strength]
3		knee NEAR/2 strength
4		"quad* strength"
5		"hamstring strength"
6		flex* NEAR/2 strength OR ext* NEAR/2 strength
7		hip NEAR/2 strength
8		calf NEAR/2 strength
9	Torque	torque
10	Isokinetic	isokinetic
11	Dynamometry	dyn?mometry
12	Quadriceps	"quadriceps muscle"
13	Hamstring	"hamstring muscle"
14		**Combine all with OR**
**Element 3 - Function**		
16	Hop	hop
17	Jump	jump
18	Functional test	function* NEAR/2 test
19		function* NEAR/2 scores
20		function* NEAR/2 outcome*
21		function* NEAR/2 measure*
22		**Combine all with OR**
**Final Search**		
24		**Combine - Element 1 AND (Element 2 OR 3)**
		Trials Only (not Cochrane Reviews)

Table 1.6: SPORTDiscus Search

Search	Term	SPORTDiscus
		**Element 1 - Anterior Cruciate Ligament**
1	anterior cruciate ligament	"anterior cruciate ligament"
2	ACL	ACL
		**Combine all with OR**
		**Element 2 - Strength**
2	Strength	"muscle strength"
3		knee W/2 strength
4		"quad* strength"
5		"hamstring strength"
6		flex* W/2 strength OR ext* W/2 strength
7		hip W/2 strength
8		calf W/2 strength
9	Torque	torque
10	Isokinetic	isokinetic
11	Dynamometry	dyn?mometry
12	Quadriceps	"quadriceps muscle"
13	Hamstring	"hamstring muscle"
14		**Combine all with OR**
		**Element 3 - Function**
16	Hop	hop
17	Jump	jump
18	Functional test	function* W/2 test
19		function* W/2 scores
20		function* W/2 outcome*
21		function* W/2 measure*
22		**Combine all with OR**
		**Final Search**
24		**Combine - Element 1 AND (Element 2 OR 3)**

Supplement 2: Risk of Bias Assessment

Table 2.1: Risk of Bias Assessment

Criteria for assessment of risk of bias domains, as outlined by Cochrane Collaboration. Criteria were pre-registered on PROSPERO.

	**Low**	**High**	**Unclear**	**N/A**
**Random Sequence Generation**	If randomisation method follows Cochrane recommendations (adequate methods computer-generated methods, use of random number tables, drawing of lots).	Trials that use pseudorandomisation (e.g. date of birth or clinical record number). All non-randomised studies.	insufficient information is available in the methods to make judgement from.	NA for observational studies
**Allocation concealment**	If the investigators responsible for patient selection are unable to suspect the allocation (e.g. central randomisation; sequential, numbered sealed opaque envelopes).	Studies with inadequate allocation concealment. All non-randomised studies.		NA for observational studies
**Blinding of patients**	If participants were unaware of their assigned intervention – e.g. if a trial was placebo controlled, a sham intervention was used, a double dummy technique was used, or if interventions described as indistinguishable.	No blinding of patients	Insufficient information provided to make judgement	NA for observational studies

**Blinding of therapist**	If blinding of therapists explicitly mentioned in text.	No mention of blinding of therapists or no information provided		NA for observational studies
**Blinding of assessor (outcome assessor)**<br><br>**(For primary outcomes of strength and or function)**	If the assessor was unaware of group allocation or blinded to leg status in nonrandomised studies (i.e. which leg suffered ACL injury)	If assessors are not blinded, or no information is provided and inadequate allocation concealment is reported.	Where studies don't provide information on assessor blinding, BUT there was adequate allocation concealment	
**Outcome measurement**	if they reference a reliable and reproducible method, along with sufficient information provided around protocol (such as instructions given, duration of contractions and rest periods between assessments).	No reliable method is referenced and inadequate methodological detail is provided to reproduce the assessment	If they lack these specific protocol details but reference a reliable method	
**Selection**	For case-control studies or studies with multiple groups we will score low risk of bias if<br>- cases and controls are taken from comparable populations (similar demographic characteristics and same exclusion criteria applied, e.g. in studies with healthy controls the only difference	If groups are recruited from different populations and/or with differing exclusion criteria beyond their knee injury status. If the groups are not representative of the entire population of interest.	Unclear risk of bias will be scored where insufficient information is provided around the selection or source of participants	Not applicable for randomised studies.

	being their ACL injury status.<br>- Groups are representative of the entire population of interest			
**Attrition**	If >80% retained in the study and comparable loss between groups	>20% dropout and or unbalanced drop-out rate across groups.	Details of drop outs not provided	Not applicable for single timepoint studies (cross-sectional).
**Analysis**	All participants need to be included in analyses (e.g. according to intention to treat principles). Measures of uncertainty are provided for all outcomes of interest.	If studies inexplicably exclude participants from analysis or measures of uncertainty (e.g. confidence intervals) are not provided.<br><br>Confounders are not controlled or discussed in analysis of non-randomised studies,		

Supplement 3 - Formulae used in data analysis

1) Approximation of ratio of means (RoM) from limb symmetry index (LSI).

The limb symmetry index is effectively a mean of ratios (MoR) Calculation of variance uses the delta method (Friedrich et al 2008; Lajeunesse et al 2011):

$$RoM = \log(\text{mean}_{LSI})$$

$$Variance_{RoM} = \frac{{SD_{LSI}}^2}{n \times {mean_{LSI}}^2}$$

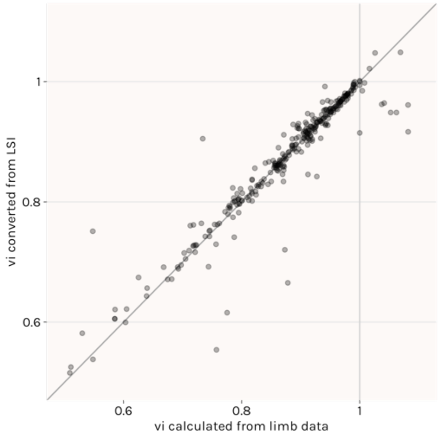

Figure 4.1: Demonstration of agreement between LSI conversion and RoM, using 318 data points from this review where a study reported data for each limb (allowing RoM calculation) as well as an LSI.

2) Conversion of range to standard deviation (from Wan et al, 2014)

$$S = \frac{b - a}{2\phi^{-1} \times (\frac{n - 0.375}{n + 0.25})}$$

Where b = range upper limit, a = range lower limit, n = sample size and ϕ^{-1} is the upper zth percentile of the standard normal distribution (computer with "qnorm" function in R)

3) *Combining means of k groups (weighted average)*

$$\bar{x}_{combined} = \frac{\sum_{i=1}^{k} n_i \cdot \bar{x}_i}{\sum_{i=1}^{k} n_i}$$

Combining SDs of k groups

$$SD_{\text{combined}} = \sqrt{\frac{\sum_{i=1}^{k}(n_i - 1) \cdot \text{SD}_i^2 + \sum_{i=1}^{k} n_i \cdot (\overline{X_i} - \overline{X_{\text{combined}}})^2}{\sum_{i=1}^{k}(n_i - 1)}}$$

Supplement 4 – Included Study Summary

Table 4.1 – Summary of included studies (n = 136).

Study	ACLR n (female)	Uninjured Control n (female)	Age, years	Body mass index, kg/m^2	Graft type	Hop tests	Hop units	Timepoint of assessment (years)	Upper limb placement during hop tests	Landing requirements during hop tests
Ahlden 2013	103 (33)		Median 25 Range 18-52	ns	HS	Single forward hop<br>Square hop	LSI<br>LSI	2.2	Free	Stable 3s
Aizawa 2022	54 (33)		20 (4.3)	21.9 (3)	mixed	Single forward hop<br>Lateral hop<br>Medial hop	cm/height<br>cm/height<br>cm/height	0.5	ns	ns
Akelman 2016	90 (48)	60 (26)	23.51 (2.46)	ns	mixed	Single forward hop	LSI	5, 7	ns	ns
Akoto 2019	82 (18)		28.5 (9.95)	ns	QT + HS - ST	Single forward hop	LSI	1.2	ns	ns
Ardern 2011	503 (163)		27.2 (8.4)	ns	HS	Single forward hop	LSI	4.4	ns	ns
Arhos 2020a	79 (39)		21.15 (7.6)	26.03 (3.31)	mixed	Single forward hop<br>Triple crossover hop<br>Triple hop<br>6m timed forward hop	LSI<br>LSI<br>LSI<br>LSI	1, 2	ns	ns
Arundale 2017#	39 (0)		23.51 (7.6)	ns	mixed	Single forward hop<br>Triple crossover hop<br>Triple hop<br>6m timed forward hop	LSI<br>LSI<br>LSI<br>LSI	1, 2	ns	ns
Barenius 2010	153 (64)		33.98 (6.96)	ns	BPTB + HS - ST	Single forward hop	LSI	8.4	ns	ns
Barie 2018	69 (27)		27	ns	QTPB	Triple hop	cm	0.6	ns	ns
Barnett 2020	65 (37)		19.38 (5.09)	24.68 (3.81)	Bridge Enhanced	Single forward hop<br>Triple hop	LSI<br>LSI	0.5, 1, 2	On hips	ns

					ACL Repair (BEAR)	6m timed forward hop<br>Triple crossover hop	LSI<br>LSI			
Bartels 2016	50 (14)		32.72 (10)	24.3 (2.66)	HS	Vertical hop	cm	0.4, 0.5	Free	ns
Baumgart 2017	50 (16)		29.2 (8.5)	26.7 (4.6)	BPTB	Vertical hop	cm	2.6	ns	ns
Beischer 2019	237 (139)		ns	ns	mixed	Vertical hop<br>Single forward hop<br>Side hop	LSI<br>LSI<br>LSI	0.3	ns	Stick landing
Bell 2016	55 (48)	73 (60)	18.97 (1.66)	ns	mixed	Single forward hop<br>Triple hop	LSI<br>LSI	2.5	ns	ns
Blakeney 2018	371 (84)	39 (10)	28 (9.9)	24 (3.3)	not stated	Single forward hop<br>Triple hop<br>Side hop<br>Triple crossover hop	LSI<br>LSI<br>LSI<br>LSI	0.5, 0.7	ns	Stick landing
Bodkin 2017	51 (36)		22.87 (4.56)	ns	mixed	Single forward hop<br>Triple hop<br>Triple crossover hop<br>6m timed forward hop	cm/height<br>cm/height<br>cm/height<br>secs	4.5	ns	ns
Boo 2020	272 (48)		26.23	24.54	mixed	Single forward hop	cm	0.4	ns	Maintain balance on landing
Bookbinder 2020	27 (20)	25 (16)	19.7 (2.18)	ns	mixed	Single forward hop	cm/cm	2.2	ns	Fixed position
Bryant 2011	39 (13)	33 (11)	28.35 (8.29)	ns	PT + HS - ST/G	Single forward hop	m/height in m	1.9	ns	Regain balance on landing
Button 2014*	21 (5)	21 (9)	29.1 (9)	ns	HS	Single forward hop	cm/height	1.4	On hips	ns
Byrne 2021	313 (0)		22.83 (5.25)	ns	BPTB	Vertical hop	LSI	0.8	ns	ns
Capin 2019#	39 (39)		18.95 (7.31)	25.01 (3.57)	mixed	Single forward hop<br>Triple crossover hop<br>Triple hop<br>6m timed forward hop	LSI<br>LSI<br>LSI<br>LSI	1, 2	On hips	ns
Carolan 2020	298 (0)		24.16 (4.6)	ns	mixed	Vertical hop	cm	0.8	ns	Maintain balance on landing

Casp 2021	144 (87)	140 (82)	21.95 (9.28)	ns	mixed	Single forward hop<br>Triple hop	m/m<br>m/m	0.5	ns	Stable 2s
Chantrelle 2023	330 (95)		26.38 (7.07)	ns	HS	Single forward hop	LSI	0.3, 0.6	ns	Stable 1s
Chen 2017	50 (0)	57 (0)	28 (6)	ns	mixed	Single forward hop	cm/leg length	3	ns	ns
Chung 2015a	75 (11)	75 (11)	27.9 (8.6)	24.7 (2.7)	HS	Single forward hop	cm	0.2, 0.5, 1, 2	ns	ns
Coquard 2022	222 (97)		37.62 (9.46)	24.46 (3.5)	HS	Single forward hop<br>Triple hop<br>Triple crossover hop	LSI<br>LSI<br>LSI	0.3	ns	Stable
Cristiani 2019	4093 (1870)		ns	ns	mixed	Single forward hop	LSI	0.5	ns	Stable
Cristiani 2020	160 (45)		28.65 (6.1)	ns	BPTB + HS	Single forward hop	LSI	0.3, 0.5, 0.7, 1, 2	Behind back	Without subsequent hops
Culvenor 2016b	97 (31)	48 (28)	Median 28 IQR 23-35	25.8 (3.9)	HS	Single forward hop	LSI	1.1	ns	Stable 2s
Curran 2020	82 (45)		20.19 (6.59)	ns	mixed	Single forward hop<br>Triple hop<br>Triple crossover hop<br>Vertical hop	LSI<br>LSI<br>LSI<br>LSI	1.3	ns	No touchdown of other limb
Dempsey 2019	60 (31)		22.4 (9.2)	ns	mixed	Single forward hop	cm/cm	0.5	ns	ns
Devitt 2022	122 (43)		24.2 (6.38)	ns	mixed	Single forward hop<br>Triple hop	LSI<br>LSI	1	ns	ns
Duckett 2021	77 (37)	76 (42)	21.9 (7.8)	ns	mixed	Single forward hop	cm/height	0.5	ns	Controlled landing
Ebert 2018	111 (38)		27.3 (9.1)	25.5 (3.6)	HS	Single forward hop<br>6m timed forward hop<br>Triple hop<br>Triple crossover hop	m<br>secs<br>m<br>m	1	ns	ns
Ebert 2019	50 (18)		26.3 (9.6)	24.8 (4)	LARS + HS	Single forward hop<br>6m timed forward hop<br>Triple hop	cm<br>secs<br>cm<br>cm	1, 2	ns	ns

						Triple crossover hop				
Ebert 2021a	50 (16)		28.3 (9.1)	24.6 (2.8)	HS	Single forward hop<br>6m timed forward hop<br>Triple hop<br>Triple crossover hop<br>Lateral hop<br>Medial hop<br>Vertical hop	LSI<br>LSI<br>LSI<br>LSI<br>LSI<br>LSI<br>LSI	0.8	ns	ns
Ebert 2022a	136 (47)		30.95 (9.94)	26.1 (3.09)	HS + LARS + HS	Single forward hop<br>6m timed forward hop<br>Triple hop<br>Triple crossover hop	LSI<br>LSI<br>LSI<br>LSI	7.8	ns	Stable
Edwards 2018	113 (38)		25.88 (8.14)	ns	HS	Single forward hop<br>6m timed forward hop<br>Triple hop<br>Triple crossover hop	LSI<br>LSI<br>LSI<br>LSI	1	ns	ns
Engelen-VanMelick 2017	97 (46)	44 (22)	36.08 (9.9)	24.47 (3.62)	BPTB + HS - ST/G	Vertical hop<br>Single forward hop<br>Side hop	cm<br>cm<br>reps	4.5	Behind back for single forward hop	ns
Ericsson 2013*	45 (ns)		ns	ns	mixed	Single forward hop<br>Square hop<br>Vertical hop	cm<br>reps<br>cm	3.1	ns	ns
Eriksson 2001	164 (ns)		ns	ns	BPTB + HS	Single forward hop<br>Triple hop	LSI<br>LSI	2.8	ns	ns
Falstrom 2017	77 (77)	77 (77)	18.4 (2.3)	22.8 (2.7)	mixed	Single forward hop<br>Side hop	cm<br>reps	1.5	ns	ns
Faltstrom 2021	117 (117)	119 (119)	20 (2)	ns	mixed	Single forward hop<br>Side hop	cm<br>reps	1.6	ns	Stable
Felix 2022	34 (7)	40 (7)	25.05 (6.82)	23.62 (2.72)	not stated	Single forward hop	LSI	1	ns	ns
Fischer 2017	169 (60)		22.35 (8.06)	22.61 (2.06)	not stated	Vertical hop	cm	0.5	ns	ns
Fleming 2013	90 (48)	60 (26)	23.51 (2.46)	ns	mixed	Single forward hop	LSI	1, 3	ns	ns
Flosadottir 2018	69 (18)		26.37 (5.19)	23.93 (2.94)	mixed	Single forward hop	LSI	5	ns	ns

Gauthier 2022	303 (91)		27 (8)	24.3 (4.2)	mixed	Triple hop<br>Side hop	m<br>reps	0.7	ns	ns
Ghalayani 2010	50 (10)		31.28<br>Range<br>29-34.5	ns	Synthetic<br>Leeds Keio +<br>PT	Single forward hop	LSI	0.5, 1,<br>2, 5	ns	ns
Gokeler 2017a	52 (14)	172 (85)	23.38<br>(3.69)	ns	mixed + HS	Single forward hop<br>Triple hop<br>Side hop	cm<br>cm<br>reps	0.6	ns	ns
Guney-Deniz 2020	67 (13)	20 (ns)	26.97<br>(4.4)	24.23<br>(1.32)	HS + TA + QT	Single forward hop	cm	1.1	ns	ns
Gupta 2018	249 (22)		Median	ns	BPTB + HS +<br>HS	Single forward hop	LSI	5.2	ns	ns
Hamrin Senorski 2017	157 (77)		22.35<br>(4.09)	ns	not stated	Vertical hop<br>Single forward hop<br>Side hop	cm/kg<br>cm/kg<br>reps/kg	0.8	ns	Controlled landing
Harput 2018	72 (0)		28 (7.6)	24.2 (4.2)	HS	Single forward hop	cm	0.5	Arms free for vertical hop	ns
Harput 2020a	91 (0)		28.55<br>(6.92)	24.87<br>(3.48)	HS	Single forward hop<br>Vertical hop	% leg length<br>cm	0.5	ns	ns
Heijne 2010	68 (32)		29.5<br>(8.02)	24.25<br>(2.64)	BPTB + HS	Single forward hop	LSI	0.6,<br>0.8, 1,<br>2	ns	ns
Heijne 2015	68 (32)		29.5<br>(8.02)	24.25<br>(2.64)	BPTB + HS	Single forward hop	LSI	5.1	ns	ns
Hu 2020	84 (41)		33.15<br>(5.18)	27.11<br>(5.01)	HS - ST/G +<br>HS - ST	Single forward hop	LSI	3.2	ns	ns
Jang 2014	67 (0)		21.88<br>(3.86)	24.47<br>(2.66)	HS	Single forward hop	cm	2.8	ns	ns
Johnston 2022	105 (23)		20 (8.64)	ns	QT + HS	Single forward hop<br>Triple crossover hop	m<br>m	0.5, 1	ns	ns
Karikis 2016	103 (33)		29.03<br>(8.88)	25.19<br>(3.08)	HS	Square hop<br>Single forward hop	reps<br>LSI	5.3	ns	ns
Kim 2011	73 (0)		28.38<br>(8.53)	ns	HS + HS<br>Allograft	Single forward hop	cm	2.7	ns	ns
Kim 2015a	80 (0)		ns	ns	not stated	Single forward hop	LSI	0.2	ns	ns
Kim 2019	185 (32)		30.59<br>(10.47)	ns	BPTB	Single forward hop	LSI	2	On hips	ns
King 2018	156 (0)		24.8 (4.8)	ns	mixed	Single forward hop	cm	0.7	On hips	ns
King 2021	115 (0)		21.61<br>(4.09)	ns	mixed	Vertical hop<br>Single forward hop	cm<br>cm	0.8	ns	ns

Kline 2018	20 (11)	45 (22)	22.8<br>Range<br>15-45	ns	mixed	Single forward hop<br>6m timed forward hop<br>Triple hop<br>Triple crossover hop	cm/leg length<br>sec/leg length<br>cm/leg length<br>cm/leg length	0.7	ns	ns
Konrads 2016	62 (17)		29.8	ns	BPTB + HS - ST	Single forward hop	cm	10	ns	ns
Koutras 2013	51 (0)		23.9 (5.67)	ns	HS	Single forward hop<br>Triple hop	LSI<br>LSI	0.2, 0.5	Behind back	ns
Kovalak 2018*	43 (0)		32.56 (4.89)	25.19 (3.22)	HS	Single forward hop	cm	8.2	ns	ns
Krishna 2020	56 (13)		26.95 (7.26)	24.7 (3.78)	HS	Single forward hop	LSI	2	ns	ns
Krych 2015	196 (100)		20.69 (6.68)	ns	BPTB	Vertical hop<br>Single forward hop<br>Triple hop	LSI<br>LSI<br>LSI	0.5	Behind back	ns
KyoungHo 2017	73 (0)		30.67 (9.81)	25.05 (3.15)	mixed	Single forward hop	LSI	0.5, 1	ns	Without losing balance
Kyritsis 2016	158 (0)		21.16 (4.18)	ns	mixed	Single forward hop<br>Triple hop<br>Triple crossover hop	LSI<br>LSI<br>LSI	0.7	ns	ns
Kyung 2015	144 (0)		29.35 (11.4)	ns	HS - ST + HS - ST/G	Single forward hop	cm	2.2	ns	ns
Laudner 2015	26 (11)	26 (11)	18.7 (3.5)	ns	BPTB	Vertical hop	cm	0.6	ns	ns
Lee 2018a	75 (ns)		27.5 (9.2)	ns	HS	Vertical hop<br>Single forward hop	cm<br>cm	3.4	ns	ns
Lee 2018b	92 (7)		30.33 (9.45)	24.52 (3.19)	HS	Single forward hop	cm	3.2	ns	ns
Lee 2019	120 (8)		27.15 (6.93)	ns	HS - ST/G Single + HS - ST/G Dual	Single forward hop	cm	3.1	ns	ns
Lee 2020	54 (11)		25.9 (9.4)	23.6 (3.7)	HS - Single bundle	Vertical hop<br>Single forward hop	LSI<br>LSI	2	ns	ns
Leister 2019	46 (26)	42 (22)	34.67 (11.03)	24.57 (3.09)	ACL repair + HS	Single forward hop<br>6m timed forward hop<br>Triple crossover hop<br>Side hop	LSI<br>LSI<br>LSI<br>LSI	1.1	ns	ns

Lui 2012	52 (0)		25.14 (4.6)	ns	mixed	Single forward hop	cm	1	ns	ns
Marigi 2022	344 (157)		26.09 (10.71)	ns	mixed	Vertical hop<br>Single forward hop<br>Triple hop	cm/height<br>cm/height<br>cm/height	0.5	Behind back	Stick landing
Markstrom 2023	47 (29)	46 (40)	24.6 (4.7)	23.7 (2.5)	HS	Single forward hop<br>Vertical hop	m<br>m	1.1	ns	ns
McGrath 2017	64 (20)	32 (16)	27.9	24.9	mixed	combined Single forward hop<br>6m timed forward hop	LSI<br>LSI	0.2, 0.3, 0.4, 0.5	ns	ns
Meierbachtol 2017	71 (44)		20.2	24.4	not stated	Single forward hop<br>Triple crossover hop<br>Triple hop<br>6m timed forward hop	cm<br>cm<br>cm<br>sec	0.5, 0.6	ns	ns
Meierbachtol 2018	58 (37)		21.2 (7.8)	ns	mixed	Single forward hop<br>Triple hop<br>Triple crossover hop<br>6m timed forward hop	LSI<br>LSI<br>LSI<br>LSI	0.7, 0.8	ns	ns
Menzer 2017	88 (39)		19.4 (3.7)	ns	mixed	Single forward hop<br>Triple hop<br>Triple crossover hop	cm/leg length<br>cm/leg length<br>cm/leg length	0.6	ns	ns
Mohtadi 2015	330 (147)		28.5 (9.77)	ns	PT + HS - QB + HS - DB	Single forward hop	LSI	1, 2	ns	ns
Moran 2022a	144 (75)		21.6 (8.1)	25.4 (4.4)	mixed	Single forward hop<br>Triple hop<br>6m timed forward hop	cm<br>cm<br>secs	0.5, 0.8	ns	Balanced landing
Moran 2022b	284 (157)		21.6 (9.5)	24.8 (4.6)	mixed	Single forward hop<br>Triple hop<br>6m timed forward hop	LSI<br>LSI<br>LSI	0.5	ns	ns
Noh 2012	71 (0)		Median 23 Range 19-45)	23.7 (2.4)	Achilles allograft	Single forward hop	LSI	2.6	ns	ns

Noh 2013a	61 (0)		Median 24 Range 18-45	23.05 (2.14)	Achilles allograft	Single forward hop	LSI	2.5	ns	ns
Noh 2013b	67 (0)		Median 23 Range 19-45)	23.7 (2.39)	Achilles allograft	Single forward hop	LSI	2.6	ns	Maintain balance on landing
Norte 2019b	77 (35)		21.55 (7.77)	ns	mixed	Single forward hop<br>Triple hop<br>Triple crossover hop<br>6m timed forward hop	cm/height<br>cm/height<br>cm/height<br>secs/cm	0.5	ns	Maintain balance on landing
Norte 2020	80 (38)	80 (43)	21.7 (7.8)	ns	mixed	Single forward hop<br>Triple hop<br>Triple crossover hop<br>6m timed forward hop	cm/height<br>cm/height<br>cm/height<br>sec/height	0.5	On hips	ns
O'Malley 2018	118 (0)	44 (0)	23.6 (5.8)	ns	BPTB	Vertical hop	cm	0.5	Free	Maintain balance on landing, no touchdown of limb
Ohji 2021	73 (31)		21 (6)	22.5 (3.8)	mixed	Single forward hop	cm/cm height	1.1	ns	Maintain balance on landing
Palmieri-Smith 2015	66 (50)		20.85 (5.99)	ns	PT	Single forward hop	LSI	0.6	ns	ns
Patterson 2020a	59 (22)	41 (14)	33 (16)	25.6 (3.6)	HS	Single forward hop<br>Triple crossover hop<br>Side hop	cm<br>cm<br>reps	1, 5.2	ns	Stable 2s
Peebles 2019b	25 (19)	30 (18)	18.7 (3)	ns	mixed	Single forward hop<br>Triple hop<br>Triple crossover hop	LSI<br>LSI<br>LSI	0.6	ns	ns
Piussi 2020	328 (208)		26.04 (9.86)	23.32 (2.81)	Mixed + mixed	Vertical hop<br>Single forward hop<br>Side hop	LSI<br>LSI<br>LSI	1	Free	Maintain balance on landing, no touchdown of limb
Pua 2017	106 (23)		26 (8)	24.2 (3.4)	mixed	Single forward hop	cm	0.5	ns	ns
Raoul 2018	182 (50)		28.7 (8.6)	23.5 (3.3)	mixed	Single forward hop<br>Triple hop	LSI<br>LSI	0.5	ns	ns

						Triple crossover hop<br>6m timed forward hop	LSI<br>LSI			
Raoul 2019	234 (61)		28.4 (8.6)	23.6 (3.3)	mixed	Single forward hop<br>Triple hop<br>Triple crossover hop<br>6m timed forward hop	LSI<br>LSI<br>LSI<br>LSI	0.5	ns	Maintain balance on landing, no touchdown of limb
Reinke 2011	69 (41)		Median 20 IQR 18-20	Median 23 IQR 22-27	not stated	Single forward hop<br>Triple hop<br>Triple crossover hop<br>6m timed forward hop	LSI<br>LSI<br>LSI<br>LSI	2.9	ns	ns
Runer 2022	90 (32)		28.05 (12.02)	23.9 (2.79)	QT + HS	Single forward hop	LSI	6.6	ns	ns
Salatkaite 2021	81 (30)		23.81 (6.87)	23.85 (2.96)	not stated	Single forward hop<br>Triple crossover hop<br>Triple hop<br>6m timed forward hop	LSI<br>LSI<br>LSI<br>LSI	0.7	Behind back	Maintain balance on landing, no touchdown of limb
SanJose 2023	89 (38)		Median 21 IQR 18-25	ns	HS	Single forward hop	LSI	0.3, 0.8	ns	ns
Siney 2010	80 (25)		29.56 (7.48)	ns	mixed	Single forward hop	LSI	0.5	Free	Controlled landing
Sipka 2019	65 (21)		28.5 (8.1)	ns	not stated	Single forward hop	cm	0.5	ns	ns
Stener 2010	64 (17)		Median 26 Range 16-38	ns	HS - ST	Single forward hop	LSI	8	ns	ns
Stropnik 2020	60 (19)		31.81 (11.63)	25.73 (3.78)	HS	Single forward hop	LSI	0.5, 1	ns	ns
Thomee 2012	82 (26)		28.24 (8.14)	ns	mixed	Vertical hop<br>Single forward hop<br>Side hop	cm<br>cm<br>reps	0.5, 1, 2	ns	Stable 2s
Thompson 2022	299 (153)		ns	ns	mixed	Single forward hop<br>Triple hop	LSI<br>LSI	0.6	ns	ns
Tourville 2014	38 (20)	32 (18)	28 (11.84)	25 (3.76)	mixed	Single forward hop	LSI	3.8	ns	ns
Tyler 2004	60 (27)		36.58 (1.46)	ns	BPTB	Single forward hop	LSI	0.2, 0.5	ns	ns

Ueda 2021	97 (46)		21.7 (8.5)	22.2 (2.9)	HS	Single forward hop	LSI	1	ns	ns
Ueda 2022	144 (62)		25.8 (11.9)	22.6 (3)	mixed	Single forward hop	LSI	1	On hips	ns
Ventura 2013	80 (29)		28.6 (7.29)	ns	HS	Vertical hop	cm	0.5, 1, 2	ns	ns
Vermesan 2014	71 (13)		28.83 (6)	24.79 (4.42)	HS	Triple hop	LSI	1	ns	ns
Vijayan 2021a	95 (18)		32.96 (9.11)	ns	HS	Single forward hop	LSI	1	ns	ns
Vijayan 2021b	69 (7)		30.22 (9.34)	ns	HS	Single forward hop	LSI	2	ns	ns
Webster 2017	2570 (908)		28.1 (10)	ns	HS	Single forward hop<br>Triple crossover hop	LSI<br>LSI	1.2	ns	ns
Webster 2018	635 (246)		28.25 (10.41)	ns	HS	Single forward hop	LSI	1	ns	Stable 2s
Welling 2018a	65 (20)		24.6 (7.05)	ns	mixed	Single forward hop	cm	0.5	ns	ns
Welling 2018b	62 (17)		24.2 (6.2)	ns	mixed	Single forward hop<br>Triple hop<br>Side hop	cm<br>cm<br>reps	0.5, 0.8	ns	ns
Welling 2020	64 (20)		27.78 (8.81)	ns	mixed	Single forward hop<br>Triple hop<br>Side hop	cm<br>cm<br>reps	0.8	ns	ns
Wellsandt 2018b	83 (27)		ns	26.6 (4.3)	mixed	Single forward hop<br>Triple crossover hop<br>Triple hop<br>6m timed forward hop	LSI<br>LSI<br>LSI<br>LSI	5.3	ns	ns
Wipfler 2011	54 (21)		ns	ns	BPTB + HS	Single forward hop	LSI	1, 8.8	ns	ns
Yuya 2020	420 (198)		23.07 (8.85)	22.35 (2.98)	HS	Single forward hop	LSI	1	ns	ns
Zhi-Cheng 2018	118 (40)		25.66 (5.18)	24.16 (3.28)	HS	Single forward hop<br>6m timed forward hop<br>Side hop<br>Triple crossover hop	LSI<br>LSI<br>LSI<br>LSI	0.5, 1	ns	ns
Zumstein 2022	100 (34)		30.4 (9.47)	ns	HS	Single forward hop<br>6m timed forward hop	LSI<br>LSI	0.8	Free	Stable 3s

n = sample size, Age & BMI are mean (SD) unless otherwise specified. ns = not stated, IQR = interquartile range, HS = hamstring, ST = semitendinosus, ST/G = semitendinosus/gracilis, BPTB = bone-patellar-tendon-bone, PT = patellar tendon, QT = quads tendon, TA =

tibialis anterior, LARS = ligament augmentation and reconstruction system, SB = single bundle, DB = double bundle, LSI = limb symmetry index, kg = kilogram, BM = body mass, s = seconds.
Note: * = study also included an ACL deficient group. # = cohort reported men and women from same RCT in separate papers.

Supplement 5 – Risk of Bias Summary

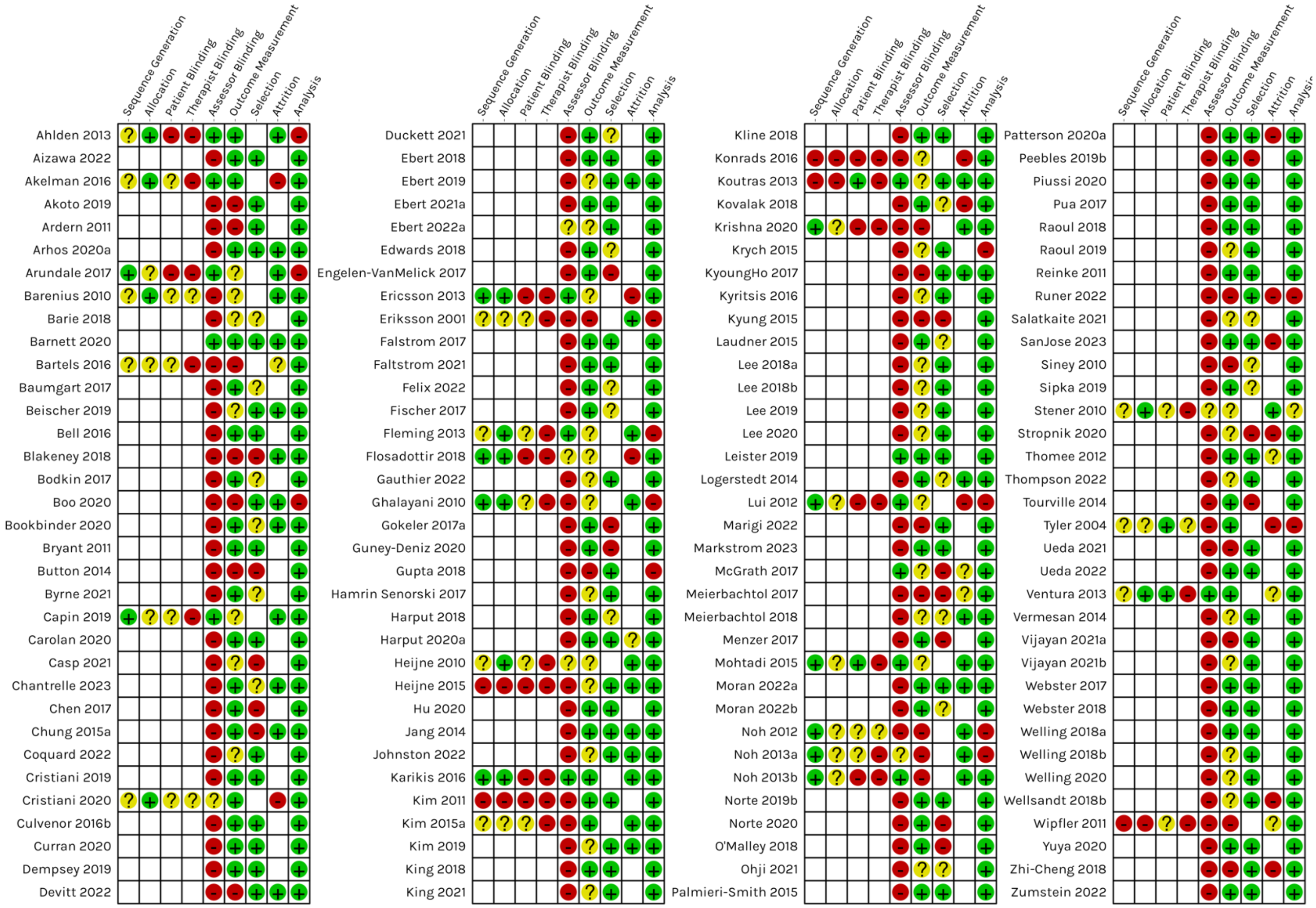

Figure 5.1: Summary of risk of bias assessment. Empty cells indicate where domains were not assessed, as they were not relevant to the study design (e.g. sequence generation for a longitudinal cohort study). Green = low risk of bias, yellow = unclear risk of bias, red = high risk of bias.

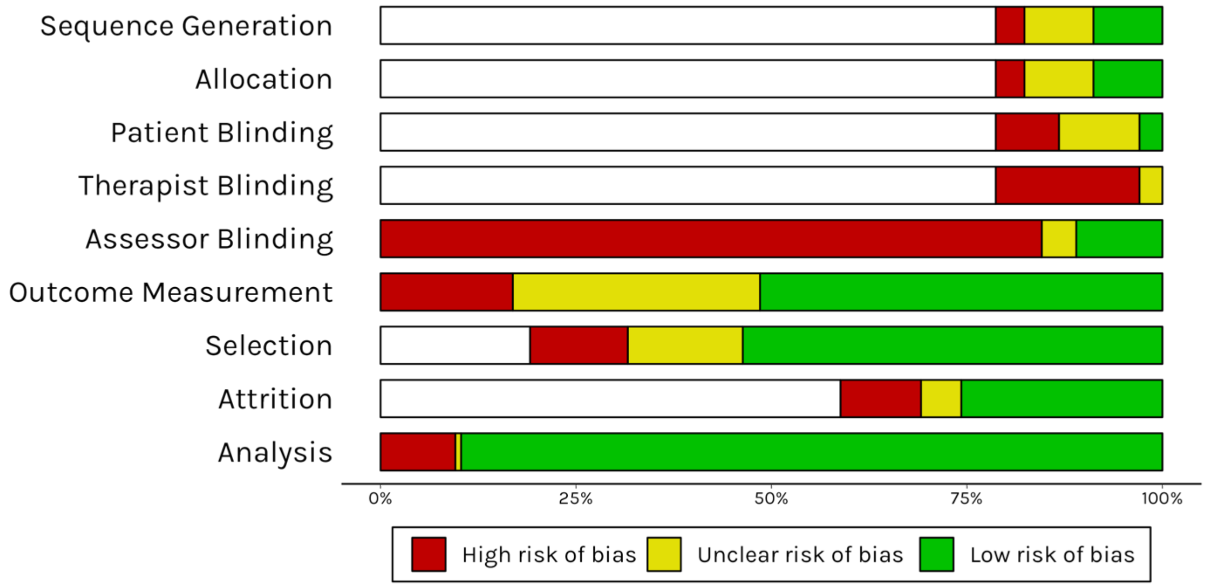

Figure 5.2: Summary of risk of bias assessment. White sections indicate where domains were not assessed, as they were not relevant to the study design (e.g. sequence generation for a longitudinal cohort study).

Supplement 6 – Details of final models presented in results

Table 6.1: Final model parameters

Model	Timepoint term	Knot positions	R^2
**Within person**			
Single forward	4 knot spline	4.1, 8.1, 13.5, 62.4	35.8
Triple forward	Log-linear	-	26.3
Triple crossover	Log-linear	-	16.0
6m timed	Log-linear	-	34.0
Side	3 knot spline	6.5, 11.1, 18.0	0.0
Vertical	Log-linear	-	36.0
**Between person**			
Single forward	Log-linear	-	7.7
Triple forward	Univariate meta-analysis only		
Triple crossover			
6m timed			
Side			
Vertical			

Supplement 7 – Results for between-person comparisons

Single Hop – Between-person comparison

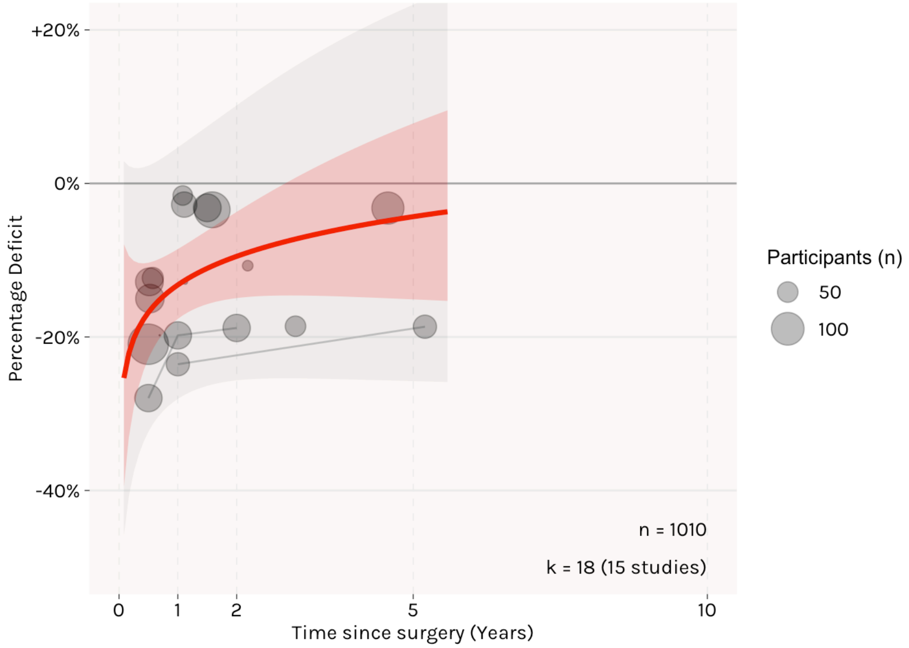

Figure 7.1: Meta-analysis of between-person comparisons of single forward hop performance. Red line and shaded region represent the estimated fit (ratio of means, expressed as percentage deficit) and 95% confidence interval, respectively, with grey shading representing the prediction interval. Grey dots represent individual cohorts with black showing linked timepoints across cohorts. n = total sample size, k = number of individual effects.

Triple Forward Hop – Between-person comparison

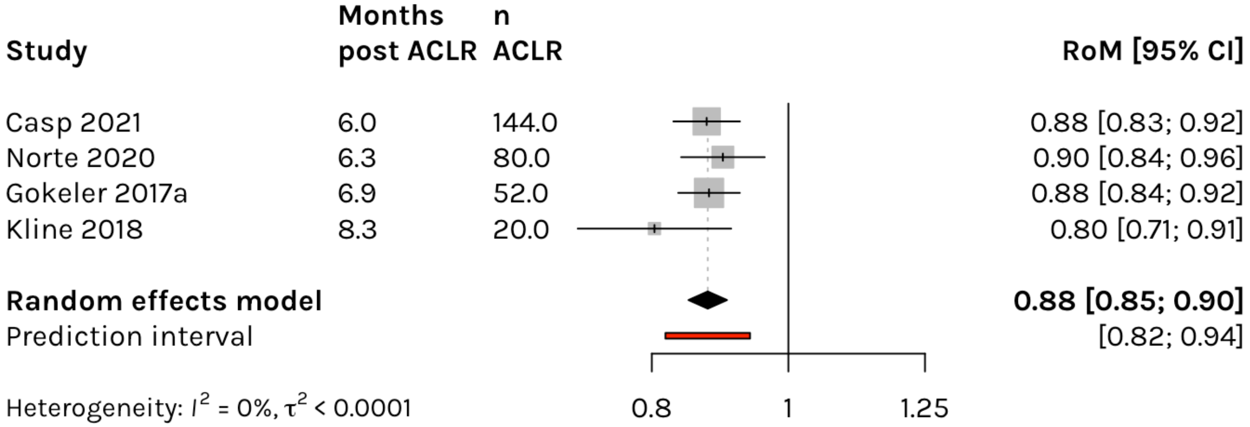

Figure 7.2: Meta analysis of between person comparisons of triple forward hop performance. ACLR = anterior cruciate ligament reconstruction; RoM = ratio of means, CI = confidence interval.

Triple Crossover Hop – Between-person comparison

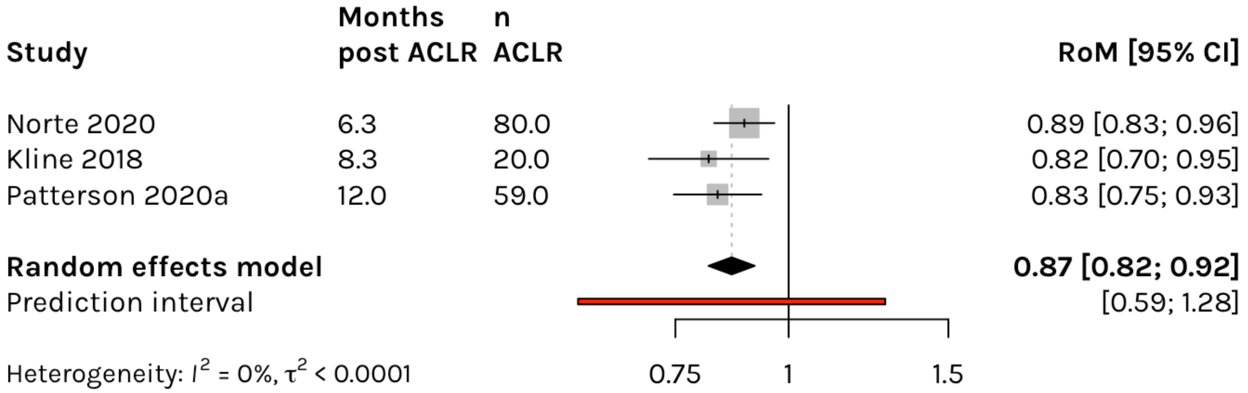

Figure 7.3: Meta analysis of between person comparisons of triple crossover hop performance. ACLR = anterior cruciate ligament reconstruction; RoM = ratio of means, CI = confidence interval.

Six Metre Timed Hop – Between-person comparison

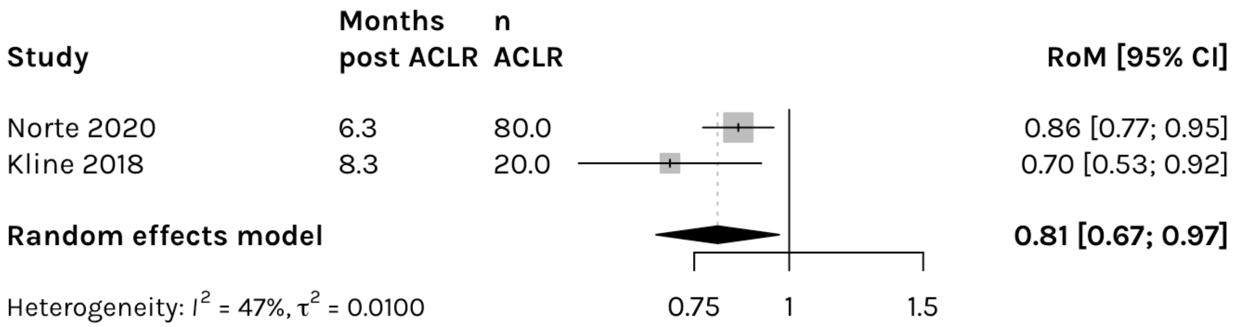

Figure 7.4: Meta analysis of between person comparisons of six metre timed hop performance. ACLR = anterior cruciate ligament reconstruction; RoM = ratio of means, CI = confidence interval.

Side Hop – Between-person comparison

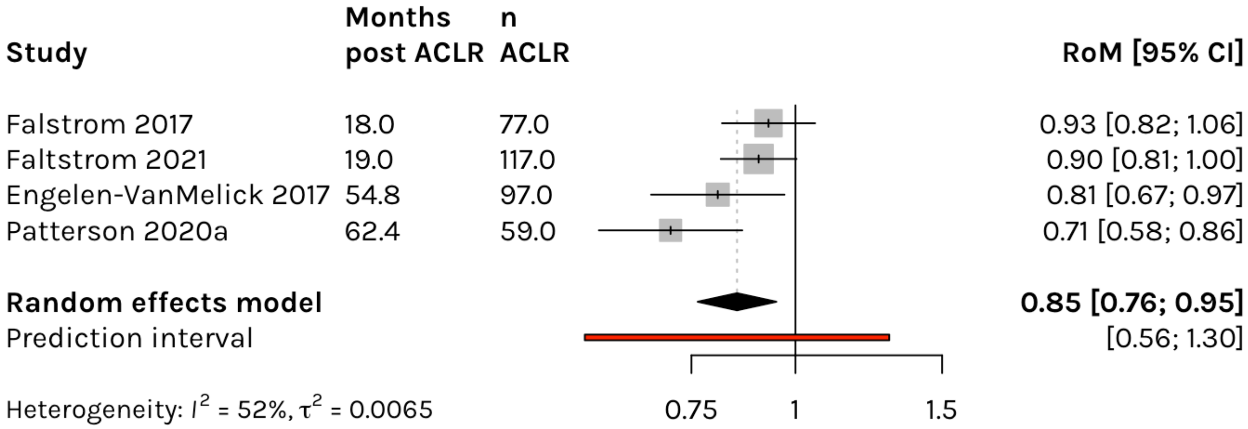

Figure 7.5: Meta analysis of between person comparisons of side hop performance. ACLR = anterior cruciate ligament reconstruction; RoM = ratio of means, CI = confidence interval.

Vertical Hop – Between-person comparison

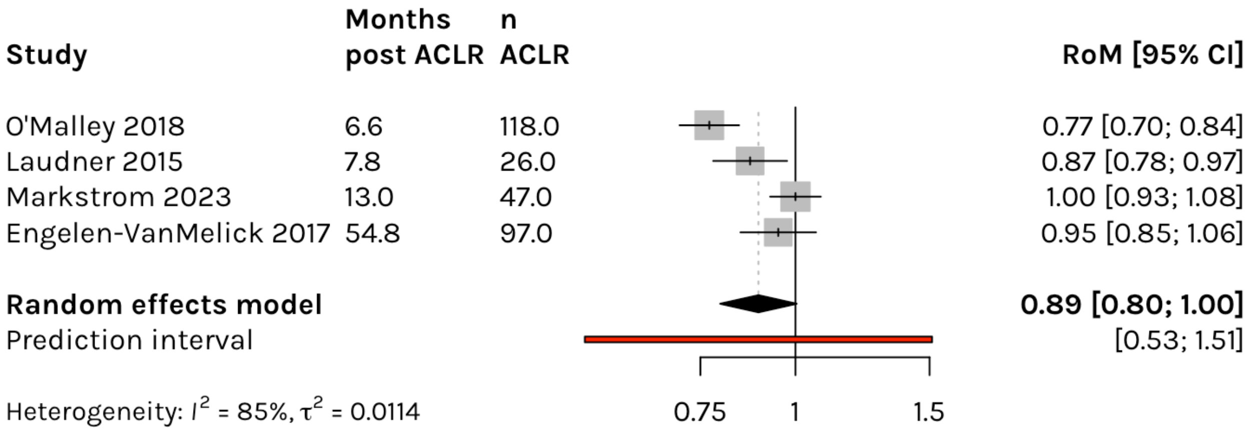

Figure 7.6: Meta analysis of between person comparisons of vertical forward hop performance. ACLR = anterior cruciate ligament reconstruction; RoM = ratio of means, CI = confidence interval.

Supplement 8 – Publication bias assessment

Table 8.1 – Results from assessment of publication bias, using a modified Egger's test with robust variance estimation as proposed by Rodgers & Pustejovsky (2021), by adding the standard error of effect as a moderator term to final models. Results present the regression estimates for the standard error term from each model.
Publication bias was not assessed for within-person comparisons of triple forward-, triple crossover-, six-metre timed-, side- and vertical hop due to lack of data (<10 studies).

	**Estimate (SE)**	**t-statistic**	**p-value**
**Within-person**			
Single Forward Hop	-3.17 (0.79)	-4.00	<0.001
Triple Forward Hop	-6.87 (1.96)	-3.50	0.006
Triple Crossover Hop	-5.89 (2.77)	-2.12	0.090
Six-metre timed hop	-5.97 (2.02)	-2.96	0.039
Side hop	-2.22 (1.94)	-1.14	0.370
Vertical hop	-0.65 (0.96)	-0.67	0.570
**Within-person**			
Single Forward Hop	-1.28 (1.11)	-1.15	0.321

SE = Standard Error

Supplement 9 – Results for other hop tests

Within-person comparisons

Two studies (Aizawa 2022 and Ebert 2021a) measured medial and lateral hop performance (maximal hop for distance in medial/lateral direction) within the first year post-ACLR. ACLR limb performance was significantly reduced for both tests, by approximately 10-15%. Square hop test performance was reported by 3 studies (Ericsson 2013, Ahlden 2013, Karikis 2016) of 2 cohorts, at follow ups >2 years post-ACLR, finding similar performance in the ACLR limb and the contralateral limb.

Between-person comparisons

No other hop tests results were reported for between-person comparisons.

Supplement 10 – Sensitivity analysis for graft type

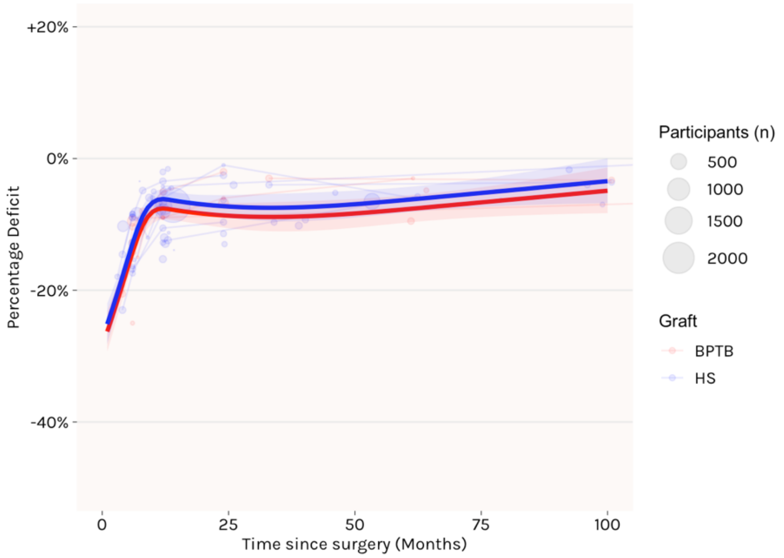

Figure 10.1: Sensitivity analysis for effect of graft type on single forward hop performance. Blue line = bone patellar tendon bone (BPTB) graft, red line = hamstring (HS) graft (any type).

Supplement 11 – Sensitivity analysis removing high risk of bias studies from within-person comparisons

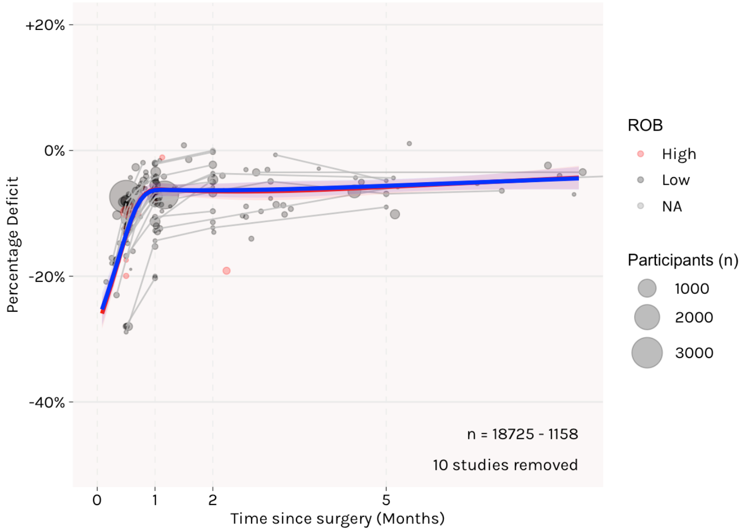

Figure 11.1 – Single forward hop sensitivity analysis removing high risk of bias studies. Blue line and shading indicate original fit from primary analysis, red line and shading indicate model fit with high risk of bias studies removed. High risk of bias studies are highlighted with red bubbles. ROB = risk of bias; n = number of participants; NA = not applicable.

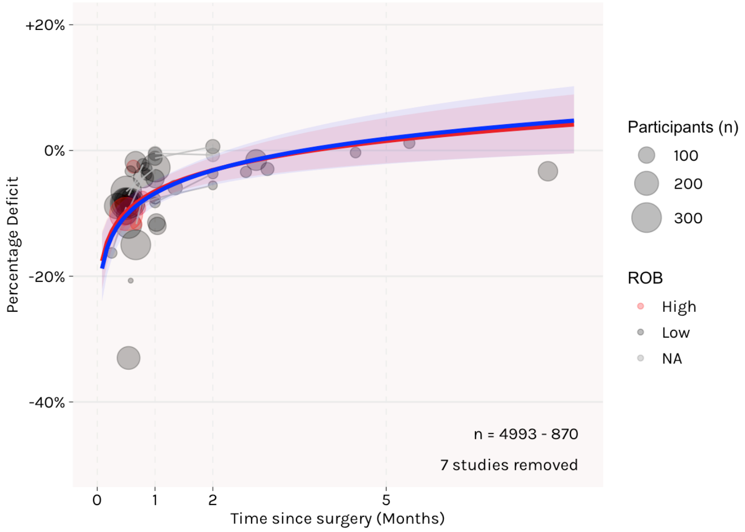

Figure 11.2 – Triple forward hop sensitivity analysis removing high risk of bias studies. Blue line and shading indicate original fit from primary analysis, red line and shading indicate model fit with high risk of bias studies removed. High risk of bias studies are highlighted with red bubbles. ROB = risk of bias; n = number of participants; NA = not applicable.

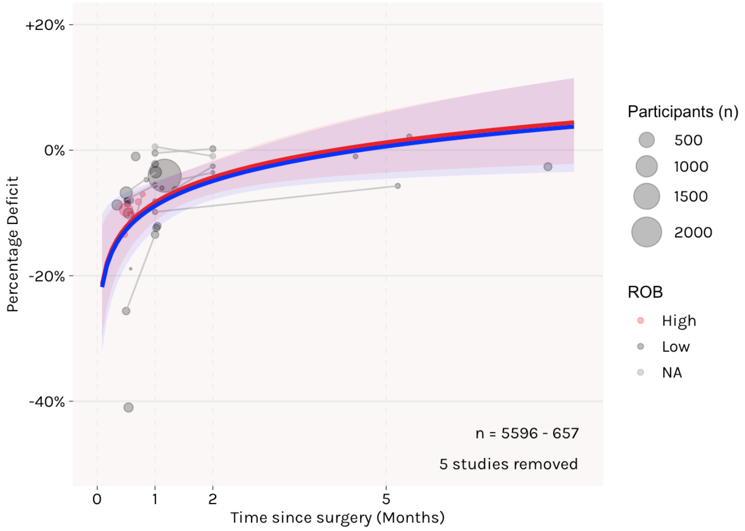

Figure 11.3 – Triple crossover hop sensitivity analysis removing high risk of bias studies. Blue line and shading indicate original fit from primary analysis, red line and shading indicate model fit with high risk of bias studies removed. High risk of bias studies are highlighted with red bubbles. ROB = risk of bias; n = number of participants; NA = not applicable.

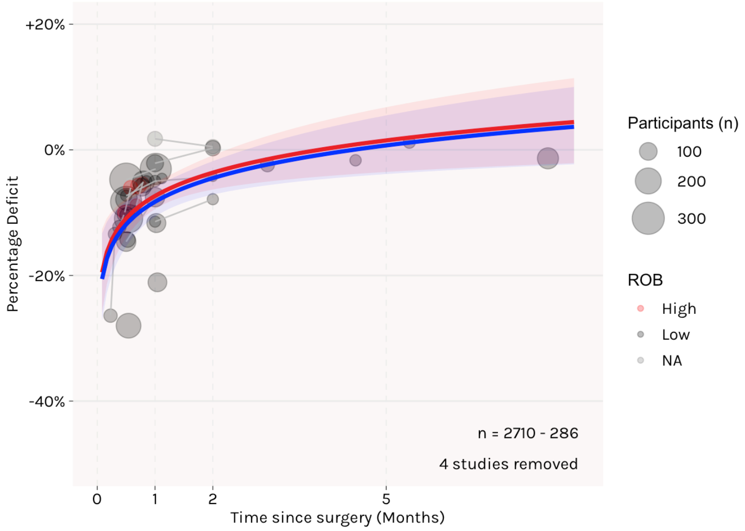

Figure 11.4 – Six metre forward hop sensitivity analysis removing high risk of bias studies. Blue line and shading indicate original fit from primary analysis, red line and shading indicate model fit with high risk of bias studies removed. High risk of bias studies are highlighted with red bubbles. ROB = risk of bias; n = number of participants; NA = not applicable.

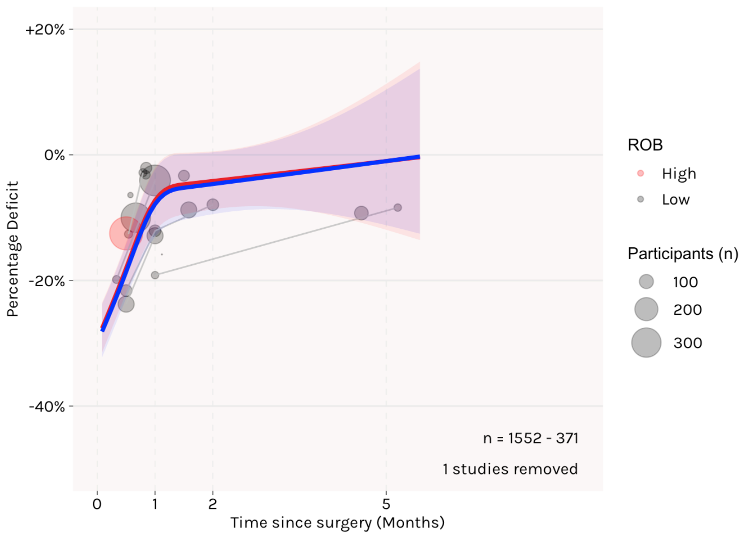

Figure 10.5 – Side hop sensitivity analysis removing high risk of bias studies. Blue line and shading indicate original fit from primary analysis, red line and shading indicate model fit with high risk of bias studies removed. High risk of bias studies are highlighted with red bubbles. ROB = risk of bias; n = number of participants; NA = not applicable.

No high risk of bias studies were included in vertical hop analysis, so no sensitivity analysis was performed.

Supplement 12 – References for included studies

1. Ahldén M, Sernert N, Karlsson J, Kartus J. A prospective randomized study comparing double- and single-bundle techniques for anterior cruciate ligament reconstruction. American journal of sports medicine. 2013;41[11]:2484-2491.

2. Aizawa J, Hirohata K, Ohji S, Ohmi T, Mitomo S, Koga H, et al. Cross-sectional study on relationships between physical function and psychological readiness to return to sport after anterior cruciate ligament reconstruction. BMC Sports Science, Medicine and Rehabilitation. 2022;14[1]:97.

3. Akelman MR, Fadale PD, Hulstyn MJ, Shalvoy RM, Garcia A, Chin KE, et al. Effect of matching or overconstraining knee laxity during anterior cruciate ligament reconstruction on knee osteoarthritis and clinical outcomes: a randomized controlled trial with 84-Month follow-up. American journal of sports medicine. 2016;44[7]:1660-1670.

4. Akoto R, Albers M, Balke M, Bouillon B, Höher J. ACL reconstruction with quadriceps tendon graft and press-fit fixation versus quadruple hamstring graft and interference screw fixation - a matched pair analysis after one year follow up. BMC musculoskeletal disorders. 2019;20[1]

5. Ardern CL, Webster KE, Taylor NF, Feller JA. Return to the preinjury level of competitive sport after anterior cruciate ligament reconstruction surgery. American journal of sports medicine. 2011;39[3]:538–43.

6. Arhos EK, Capin JJ, Ito N, Snyder-Mackler L. Functional measures do not differ in late stage rehabilitation after anterior cruciate ligament reconstruction according to mechanism of injury. International Journal of Sports Physical Therapy. 2020;15[5]:744–54.

7. Arundale AJH, Cummer K, Capin JJ, Zarzycki R, Snyder-Mackler L. Report of the clinical and functional primary outcomes in men of the ACL-SPORTS trial: similar outcomes in men receiving secondary prevention with and without perturbation training 1 and 2 years after ACL reconstruction. Clinical orthopaedics and related research. 2017;475[10]:2523-2534.

8. Barenius B, Nordlander M, Ponzer S, Tidermark J, Eriksson K. Quality of life and clinical outcome after anterior cruciate ligament reconstruction using patellar tendon graft or quadrupled semitendinosus graft: an 8-year follow-up of a randomized controlled trial. American journal of sports medicine. 2010;38[8]:1533-1541.

9. Barié A, Köpf M, Jaber A, Moradi B, Schmitt H, Huber J, et al. Long-term follow-up after anterior cruciate ligament reconstruction using a press-fit quadriceps tendon-patellar bone autograft. BMC musculoskeletal disorders. 2018;19[1]:368–368.

10. Barnett S, Badger GJ, Kiapour A, Yen YM, Henderson R, Freiberger C, et al. Females have earlier muscle strength and functional recovery after bridge-enhanced anterior cruciate ligament repair. Tissue Engineering - Part A. 2020;26[13–14]:702–11.

11. Bartels T, Proeger S, Brehme K, Pyschik M, Delank KS, Schulze S, et al. The SpeedCourt system in rehabilitation after reconstruction surgery of the anterior cruciate ligament (ACL). Archives of orthopaedic and trauma surgery. 2016;136[7]:957-966.

12. Baumgart C, Schubert M, Hoppe M, Gokeler A, Freiwald J, Hoppe MW. Do ground reaction forces during unilateral and bilateral movements exhibit compensation strategies following ACL reconstruction? Knee surgery, sports traumatology, arthroscopy. 2017;25[5]:1385–94.

13. Beischer S, Hamrin Senorski E, Thomeé C, Samuelsson K, Thomeé R. Knee strength, hop performance and self-efficacy at 4 months are associated with symmetrical knee muscle function in young athletes 1 year after an anterior cruciate ligament reconstruction. BMJ Open Sport and Exercise Medicine. 2019;5[1].

14. Bell DR, Trigsted SM, Post EG, Walden CE. Hip strength in patients with quadriceps strength deficits after ACL reconstruction. Medicine and science in sports and exercise. 2016;48[10]:1886-1892.

15. Blakeney WG, Ouanezar H, Rogowski I, Vigne G, Guen ML, Fayard JM, et al. Validation of a composite test for assessment of readiness for return to sports after anterior cruciate ligament reconstruction: The K-STARTS test. Sports Health: A Multidisciplinary Approach. 2018;10[6]:515–22.

16. Bodkin S, Goetschius J, Hertel J, Hart J. Relationships of muscle function and subjective knee function in patients after ACL reconstruction. Orthopaedic journal of sports medicine. 2017;5[7]:1-Jul.

17. Boo HC, Howe TS, Koh JSB, Boo HC, Howe TS. Effect of leg dominance on early functional outcomes and return to sports after anterior cruciate ligament reconstruction. Journal of Orthopaedic Surgery (10225536). 2020;28[1]:1-Aug.

18. Bookbinder H, Slater LV, Simpson A, Hertel J, Hart JM. Single-leg jump performance before and after exercise in healthy and anterior cruciate ligament reconstructed individuals. Journal of sport rehabilitation. 2020;29[7]:879–85.

19. Bryant AL, Clark RA, Pua YH. Morphology of hamstring torque-time curves following ACL injury and reconstruction: mechanisms and implications. Journal of orthopaedic research : official publication of the Orthopaedic Research Society. 2011;29[6]:907–14.

20. Button K, Roos PE, van Deursen RWM. Activity progression for anterior cruciate ligament injured individuals. Clinical Biomechanics. 2014;29[2]:206–12.

21. Byrne L, King E, Mc Fadden C, Jackson M, Moran R, Daniels K. The effect of meniscal pathology and management with ACL reconstruction on patient-reported outcomes, strength, and jump performance ten months post-surgery. The Knee. 2021;32:72–9.

22. Capin JJ, Failla M, Zarzycki R, Dix C, Johnson JL, Smith AH, et al. Superior 2-Year functional outcomes among young female athletes after ACL reconstruction in 10 return-to-sport training sessions: comparison of ACL-SPORTS randomized controlled trial with delaware-oslo and MOON cohorts. Orthopaedic journal of sports medicine. 2019;7[8].

23. Carolan D, King E, Richter C, Franklyn-Miller A, Moran R, Jackson M. Differences in strength, patient-reported outcomes, and return-to-play rates between athletes with primary versus revision ACL reconstruction at 9 months after surgery. Orthopaedic journal of sports medicine. 2020;8[9]:1-Jul.

24. Casp AJ, Bodkin SG, Gwathmey FW, Werner BC, Miller MD, Diduch DR, et al. Effect of meniscal treatment on functional outcomes 6 months after anterior cruciate ligament reconstruction. Orthopaedic Journal of Sports Medicine. 2021;9[10].

25. Chantrelle M, Menu P, Crenn V, Grondin J, Daley P, Louguet B, et al. Consequences of anterior knee pain after anterior cruciate ligament reconstruction: A 2015-2020 cohort study. PloS one. 2023;18[1]:e0280146.

26. Chen W- L, Chen Y- T, Huang S- Y, Yang C- Y, Wu C- D, Chang C- W. Landing strategies focusing on the control of tibial rotation in the initial contact period of one-leg forward hops. Scandinavian journal of medicine & science in sports. 2017;27[8]:832–41.

27. Chung KS, Ha JK, Yeom CH, Ra HJ, Lim JW, Kwon MS, et al. Are muscle strength and function of the uninjured lower limb weakened after anterior cruciate ligament injury? American journal of sports medicine. 2015;43[12]:3013–21.

28. Coquard M, Carrozzo A, Saithna A, Vigne G, Le Guen M, Fournier Y, et al. Anterolateral ligament reconstruction does not delay functional recovery, rehabilitation, and return to sport after anterior cruciate ligament reconstruction: A matched-pair analysis from the SANTI (scientific ACL network international) study group. Arthroscopy, Sports Medicine, and Rehabilitation. 2022;4[1]:e9–16.

29. Cristiani R, Mikkelsen C, Wange P, Olsson D, Stalman A, Engstrom B. Autograft type affects muscle strength and hop performance after ACL reconstruction. A randomised controlled trial comparing patellar tendon and hamstring tendon

autografts with standard or accelerated rehabilitation. Knee surgery, sports traumatology, arthroscopy : official journal of the ESSKA. 2020;

30. Cristiani R, Mikkelsen C, Forssblad M, Engström B, Stålman A. Only one patient out of five achieves symmetrical knee function 6 months after primary anterior cruciate ligament reconstruction. Knee surgery, sports traumatology, arthroscopy. 2019;27[11]:3461–70.

31. Culvenor AG, Alexander BC, Clark RA, Collins NJ, Ageberg EVA, Morris HG, et al. Dynamic single-leg postural control is impaired bilaterally following anterior cruciate ligament reconstruction: Implications for reinjury risk. Journal of Orthopaedic & Sports Physical Therapy. 2016;46[5]:357–64.

32. Curran MT, Bedi A, Kujawa M, Palmieri-Smith R. A cross-sectional examination of quadriceps strength, biomechanical function, and functional performance from 9 to 24 months after anterior cruciate ligament reconstruction. American journal of sports medicine. 2020;48[10]:2438–46.

33. Dempsey IJ, Norte GE, Hall M, Goetschius J, Slater LV, Cancienne JM, et al. Relationship between physical therapy characteristics, surgical procedure, and clinical outcomes in patients after ACL reconstruction. Journal of sport rehabilitation. 2019;28[2]:171–9.

34. Devitt BM, Klemm HJ, Kirby J, Batty LM, Webster KE, Whitehead TS, et al. Effect of radiological evidence of kaplan fiber injury on the clinical and functional outcomes after acute anterior cruciate ligament reconstruction. The American journal of sports medicine. 2022;50[13]:3557–64.

35. Duckett TR, Fox C, Hart JM, Norte GE. Rationale for a parsimonious measure of subjective knee function after ACL reconstruction: A rasch analysis. Journal of athletic training. 2021;

36. Ebert JR, Annear PT. ACL reconstruction using autologous hamstrings augmented with the ligament augmentation and reconstruction system provides good clinical scores, high levels of satisfaction and return to sport, and a low retear rate at 2 years. Orthopaedic journal of sports medicine. 2019;7[10]

37. Ebert JR, Du Preez L, Furzer B, Edwards P, Joss B. Which hop tests can best identify functional limb asymmetry in patients 9-12 months after anterior cruciate ligament reconstruction employing a hamstrings tendon autograft? International Journal of Sports Physical Therapy. 2021;16[2]:393–403.

38. Ebert JR, Edwards P, Yi L, Joss B, Ackland T, Carey-Smith R, et al. Strength and functional symmetry is associated with post-operative rehabilitation in patients following anterior cruciate ligament reconstruction. Knee surgery, sports traumatology, arthroscopy. 2018;26[8]:2353–61.

39. Ebert JR, Nairn R, Breidahl W, Annear PT. Double-bundle anterior cruciate ligament reconstruction using autologous hamstrings with LARS augmentation demonstrates comparable outcomes to hamstrings alone, without evidence of synovitis or early osteoarthritis. Knee surgery, sports traumatology, arthroscopy : official journal of the ESSKA. 2022;30[7]:2320–8.

40. Edwards PK, Ebert JR, Joss B, Ackland T, Annear P, Buelow JU, et al. Patient characteristics and predictors of return to sport at 12 months after anterior cruciate ligament reconstruction: The importance of patient age and postoperative rehabilitation. Orthopaedic journal of sports medicine. 2018;6[9]:1-Jan.

41. Engelen-Van Melick N, Cingel R, Tienen T, Nijhuis-Van Der Sanden M, van Cingel REH, van Tienen TG, et al. Functional performance 2-9 years after ACL reconstruction: cross-sectional comparison between athletes with bone-patellar tendon-bone, semitendinosus/gracilis and healthy controls. Knee surgery, sports traumatology, arthroscopy. 2017;25[5]:1412–23.

42. Ericsson YB, Roos EM, Frobell RB. Lower extremity performance following ACL rehabilitation in the KANON-trial: impact of reconstruction and predictive value at 2 and 5 years. British journal of sports medicine. 2013;47[15]:980-985.

43. Eriksson K, Anderberg P, Hamberg P, Löfgren AC, Bredenberg M, Westman I, et al. A comparison of quadruple semitendinosus and patellar tendon grafts in reconstruction of the anterior cruciate ligament. Journal of bone and joint surgery British volume. 2001;83[3]:348-354.

44. Fältström A, Hägglund M, Kvist J. Functional performance among active female soccer players after unilateral primary anterior cruciate ligament reconstruction compared with knee-healthy controls. American journal of sports medicine. 2017;45[2]:377–85.

45. Faltstrom A, Kvist J, Bittencourt NFN, Mendonca LD, Hagglund M. Clinical risk profile for a second anterior cruciate ligament injury in female soccer players after anterior cruciate ligament reconstruction. The American journal of sports medicine. 2021;49[6]:1421–30.

46. Felix ECR, Alonso AC, Brech GC, Fernandes TL, Almeida AMD, Luna NMS, et al. Is 12 months enough to reach function after athletes' ACL reconstruction: a prospective longitudinal study. Clinics. 2022;77:100092.

47. Fischer F, Blank C, Dünnwald T, Gföller P, Herbst E, Hoser C, et al. Isokinetic extension strength is associated with single-leg vertical jump height. Orthopaedic journal of sports medicine. 2017;5[11]:1-Jan.

48. Fleming BC, Fadale PD, Hulstyn MJ, Shalvoy RM, Oksendahl HL, Badger GJ, et al. The effect of initial graft tension after anterior cruciate ligament reconstruction: a

randomized clinical trial with 36-month follow-up. American journal of sports medicine. 2013;41[1]:25-34.

49. Flosadottir V, Frobell R, Roos EM, Ageberg E. Impact of treatment strategy and physical performance on future knee-related self-efficacy in individuals with ACL injury. BMC musculoskeletal disorders. 2018;19[1]:50.

50. Gauthier M, Le TN, Wehn A, Joseph S, Tscholl PM. Subjective knee apprehension is not associated to physical parameters 6-12 months after anterior cruciate ligament reconstruction. Journal of Experimental Orthopaedics. 2022;9[1]:110.

51. Ghalayini SR, Helm AT, Bonshahi AY, Lavender A, Johnson DS, Smith RB. Arthroscopic anterior cruciate ligament surgery: results of autogenous patellar tendon graft versus the Leeds-Keio synthetic graft five year follow-up of a prospective randomised controlled trial. The Knee. 2010;17[5]:334-339.

52. Gokeler A, Welling W, Benjaminse A, Lemmink K, Seil R, Zaffagnini S. A critical analysis of limb symmetry indices of hop tests in athletes after anterior cruciate ligament reconstruction: A case control study. Orthopaedics & traumatology, surgery & research : OTSR. 2017;103[6]:947–51.

53. Guney-Deniz H, Harput G, Kaya D, Nyland J, Doral MN. Quadriceps tendon autograft ACL reconstructed subjects overshoot target knee extension angle during active proprioception testing. Knee surgery, sports traumatology, arthroscopy. 2020;28[2]:645–52.

54. Gupta R, Sood M, Malhotra A, Masih GD, Kapoor A, Raghav M, et al. Low re-rupture rate with BPTB autograft and semitendinosus gracilis autograft with preserved insertions in ACL reconstruction surgery in sports persons. Knee surgery, sports traumatology, arthroscopy. 2018;26[8]:2381–8.

55. Hamrin Senorski E, Samuelsson K, Thomeé C, Beischer S, Karlsson J, Thomeé R. Return to knee-strenuous sport after anterior cruciate ligament reconstruction: a report from a rehabilitation outcome registry of patient characteristics. Knee surgery, sports traumatology, arthroscopy. 2017;25[5]:1364–74.

56. Harput G, Guney-Deniz H, Ozer H, Baltaci G, Mattacola C. Higher body mass index adversely affects knee function after anterior cruciate ligament reconstruction in individuals who are recreationally active. Clinical journal of sport medicine : official journal of the Canadian Academy of Sport Medicine. 2020;30[6]:e194–200.

57. Harput G, Ozer H, Baltaci G, Richards J. Self-reported outcomes are associated with knee strength and functional symmetry in individuals who have undergone anterior cruciate ligament reconstruction with hamstring tendon autograft. The Knee. 2018;25[5]:757–64.

58. Heijne A, Werner S. A 2-year follow-up of rehabilitation after ACL reconstruction using patellar tendon or hamstring tendon grafts: a prospective randomised outcome study. Knee surgery, sports traumatology, arthroscopy. 2010;18[6]:805-813.

59. Heijne A, Hagströmer M, Werner S. A two- and five-year follow-up of clinical outcome after ACL reconstruction using BPTB or hamstring tendon grafts: a prospective intervention outcome study. Knee surgery, sports traumatology, arthroscopy. 2015;23[3]:799–807.

60. Hu A, Lawton CD, Nelson P, Selley RS, Sweeney P, Tuttle J, et al. Assessment of flexion strength following single- versus double-hamstring tendon harvest for anterior cruciate ligament reconstruction. Arthroscopy: The Journal of Arthroscopy & Related Surgery. 2020;36[5]:1409–16.

61. Jang SH, Kim JG, Ha JK, Wang BG, Yang SJ. Functional performance tests as indicators of returning to sports after anterior cruciate ligament reconstruction. Knee. 2014;21[1]:95–101.

62. Johnston PT, Feller JA, McClelland JA, Webster KE. Knee strength deficits following anterior cruciate ligament reconstruction differ between quadriceps and hamstring tendon autografts. Knee surgery, sports traumatology, arthroscopy : official journal of the ESSKA. 2022;30[4]:1300–10.

63. Karikis I, Desai N, Sernert N, Rostgard-Christensen L, Kartus J. Comparison of anatomic double- and single-bundle techniques for anterior cruciate ligament reconstruction using hamstring tendon autografts: a prospective randomized study with 5-Year clinical and radiographic follow-up. American journal of sports medicine. 2016;44[5]:1225-1236.

64. Kim DK, Hwang JH, Park WH. Effects of 4 weeks preoperative exercise on knee extensor strength after anterior cruciate ligament reconstruction. Journal of Physical Therapy Science. 2015;27[9]:2693–6.

65. Kim JG, Yang SJ, Lee YS, Shim JC, Ra HJ, Choi JY. The effects of hamstring harvesting on outcomes in anterior cruciate ligament reconstructed patients: A comparative study between hamstring-harvested and -unharvested patients. Arthroscopy - journal of arthroscopic and related surgery. 2011;27[9]:1226–34.

66. Kim SJ, Choi CH, Chun YM, Kim SH, Lee SK, Jung WS, et al. Anterior cruciate ligament reconstruction using bone-patellar tendon-bone autograft with remnant preservation: Comparison of outcomes according to the amount of remnant tissue. The journal of knee surgery. 2019;32[9]:847–59.

67. King E, Richter C, Daniels KAJ, Franklyn-Miller A, Falvey E, Myer GD, et al. Can biomechanical testing after anterior cruciate ligament reconstruction identify

athletes at risk for subsequent ACL injury to the contralateral uninjured limb? The American journal of sports medicine. 2021;49[3]:609–19.

68. King E, Richter C, Franklyn-Miller A, Daniels K, Wadey R, Moran R, et al. Whole-body biomechanical differences between limbs exist 9 months after ACL reconstruction across jump/landing tasks. Scandinavian journal of medicine & science in sports. 2018;28[12]:2567–78.

69. Kline PW, Noehren B, Burnham J, Yonz M, Johnson D, Ireland ML. Hip external rotation strength predicts hop performance after anterior cruciate ligament reconstruction. Knee surgery, sports traumatology, arthroscopy. 2018;26[4]:1137–44.

70. Konrads C, Reppenhagen S, Plumhoff P, Hoberg M, Rudert M, Barthel T. No significant difference in clinical outcome and knee stability between patellar tendon and semitendinosus tendon in anterior cruciate ligament reconstruction. Archives of orthopaedic and trauma surgery. 2016;136[4]:521-525.

71. Koutras G, Papadopoulos P, Terzidis IP, Gigis I, Pappas E. Short-term functional and clinical outcomes after ACL reconstruction with hamstrings autograft: transtibial versus anteromedial portal technique. Knee surgery, sports traumatology, arthroscopy. 2013;21[8]:1904–9.

72. Kovalak E, Atay T, Cetin C, Atay IM, Serbest MO. Is ACL reconstruction a prerequisite for the patients having recreational sporting activities? Acta orthopaedica ET traumatologica turcica. 2018;52[1]:37–43.

73. Krishna L, Chan CX, Lokaiah L, Chinnasamy D, Goyal S, Wang M, et al. Five-strand versus four-strand hamstring autografts in anterior cruciate ligament reconstruction - A prospective randomized controlled study. Arthroscopy. 2020;

74. Krych A, Arutyunyan G, Kuzma S, Levy B, Dahm D, Stuart M. Adverse effect of femoral nerve blockade on quadriceps strength and function after ACL reconstruction. The journal of knee surgery. 2015;28[1]:83–8.

75. Kyoung Ho S, Joong Hoon L, Seung-Yeol L, Chong-Bum C, Jae-Young L. Longitudinal changes in knee muscles isokinetic strength and dynamic performance in patients following reconstruction of the anterior cruciate ligament. Isokinetics & Exercise Science. 2017;25[4]:249–57.

76. Kyritsis P, Bahr R, Landreau P, Miladi R, Witvrouw E. Likelihood of ACL graft rupture: not meeting six clinical discharge criteria before return to sport is associated with a four times greater risk of rupture. British journal of sports medicine. 2016;50[15]:946–51.

77. Kyung HS, Lee HJ, Oh CW, Hong HP. Comparison of results after anterior cruciate ligament reconstruction using a four-strand single semitendinosus or a semitendinosus and gracilis tendon. Knee surgery, sports traumatology, arthroscopy. 2015;23[11]:3238–43.

78. Laudner K, Evans D, Wong R, Allen A, Kirsch T, Long B, et al. Relationship between isokinetic knee strength and jump characteristics following anterior cruciate ligament reconstruction. International Journal of Sports Physical Therapy. 2015;10[3]:272–80.

79. Lee DW, Kim JG, Kim HT, Cho SI. Evaluation of anterolateral ligament healing after anatomic anterior cruciate ligament reconstruction. American journal of sports medicine. 2020;48[5]:1078–87.

80. Lee DW, Shim JC, Yang SJ, Cho SI, Kim JG. Functional effects of single semitendinosus tendon harvesting in anatomic anterior cruciate ligament reconstruction: Comparison of single versus dual hamstring harvesting. Clinics in orthopedic surgery. 2019;11[1]:60–72.

81. Lee DW, Yang SJ, Cho SI, Lee JH, Kim JG. Single-leg vertical jump test as a functional test after anterior cruciate ligament reconstruction. The Knee. 2018;25[6]:1016–26.

82. Lee DW, Yeom CH, Kim DH, Kim TM, Kim JG. Prevalence and predictors of patellofemoral osteoarthritis after anterior cruciate ligament reconstruction with hamstring tendon autograft. Clinics in orthopedic surgery. 2018;10[2]:181–90.

83. Leister I, Kulnik ST, Kindermann H, Ortmaier R, Barthofer J, Vasvary I, et al. Functional performance testing and return to sport criteria in patients after anterior cruciate ligament injury 12–18 months after index surgery: A cross-sectional observational study. Physical therapy in sport. 2019;37:1-Sep.

84. Logerstedt D, DI Stasi S, Grindem H, Lynch A, Eitzen I, Engebretsen L, et al. Self-reported knee function can identify athletes who fail return-to-activity criteria up to 1 year after anterior cruciate ligament reconstruction: A delaware-oslo ACL cohort study. Journal of Orthopaedic & Sports Physical Therapy. 2014;44[12]:914–23.

85. Lui PP, Cheng YY, Yung SH, Hung AS, Chan KM. A randomized controlled trial comparing bone mineral density changes of three different ACL reconstruction techniques. The Knee. 2012;19[6]:779-785.

86. Marigi EM, Hale RF, Bernard CD, Bates N, Stuart MJ, Hewett TE, et al. Are 6-Month functional and isokinetic testing measures risk factors for second anterior cruciate ligament injuries at long term follow-up? Journal of Knee Surgery. 2022;

87. Markstrom JL, Naili JE, Hager CK. A minority of athletes pass symmetry criteria in a series of hop and strength tests irrespective of having an ACL reconstructed knee or being noninjured. Sports health. 2023;15[1]:45–51.

88. McGrath TM, Waddington G, Scarvell JM, Ball N, Creer R, Woods K, et al. An ecological study of anterior cruciate ligament reconstruction, part 2. Orthopaedic journal of sports medicine. 2017;5[2]:1-Jan.

89. Meierbachtol A, Rohman E, Paur E, Bottoms J, Tompkins M. Quantitative improvements in hop test scores after a 6-Week neuromuscular training program. Sports Health: A Multidisciplinary Approach. 2017;9[1]:22–9.

90. Meierbachtol A, Yungtum W, Paur E, Bottoms J, Chmielewski TL. Psychological and functional readiness for sport following advanced group training in patients with anterior cruciate ligament reconstruction. Journal of Orthopaedic & Sports Physical Therapy. 2018;48[11]:864–72.

91. Menzer H, Slater LV, Diduch D, Miller M, Norte G, Goetschius J, et al. The utility of objective strength and functional performance to predict subjective outcomes after anterior cruciate ligament reconstruction. Orthopaedic journal of sports medicine. 2017;5[12]:1-Jan.

92. Mohtadi N, Chan D, Barber R, Oddone Paolucci E. A randomized clinical trial comparing patellar tendon, hamstring tendon, and double-bundle ACL reconstructions: patient-Reported and clinical outcomes at a minimal 2-Year follow-up. Clinical journal of sport medicine. 2015;25[4]:321-331.

93. Moran TE, Ignozzi AJ, Burnett Z, Bodkin S, Hart JM, Werner BC. Deficits in contralateral limb strength can overestimate limb symmetry index after anterior cruciate ligament reconstruction. Arthroscopy, Sports Medicine, and Rehabilitation. 2022;4[5]:e1713–9.

94. Moran TE, Ignozzi AJ, Taleghani ER, Bruce AS, Hart JM, Werner BC. Flexible versus rigid reaming systems for independent femoral tunnel reaming during ACL reconstruction: Minimum 2-Year clinical outcomes. Orthopaedic Journal of Sports Medicine. 2022;10[3].

95. Noh JH, Roh YH, Yang BG, Yi SR, Lee SY. Femoral tunnel position on conventional magnetic resonance imaging after anterior cruciate ligament reconstruction in young men: transtibial technique versus anteromedial portal technique. Arthroscopy. 2013;29[5]:882-890.

96. Noh JH, Yang BG, Yi SR, Roh YH, Lee JS. Hybrid tibial fixation for anterior cruciate ligament reconstruction with Achilles tendon allograft. Arthroscopy. 2012;28[10]:1540-1546.

97. Noh JH, Yang BG, Yi SR, Roh YH, Lee JS. Single-bundle anterior cruciate ligament reconstruction in active young men using bone-tendon achilles allograft versus free tendon achilles allograft. Arthroscopy. 2013;29[3]:507-513.

98. Norte GE, Goetschius JW, Slater LV, Hart JM. Influence of patient demographics and surgical characteristics on pass rates of return-to-activity tests in anterior cruciate ligament-reconstructed patients before physician clearance. Clinical journal of sport medicine : official journal of the Canadian Academy of Sport Medicine. 2020;

99. Norte GE, Solaas H, Saliba SA, Goetschius J, Slater LV, Hart JM. The relationships between kinesiophobia and clinical outcomes after ACL reconstruction differ by self-reported physical activity engagement. Physical therapy in sport. 2019;40:1-Sep.

100. O'Malley E, Richter C, King E, Strike S, Moran K, Franklyn-Miller A, et al. Countermovement jump and isokinetic dynamometry as measures of rehabilitation status after anterior cruciate ligament reconstruction. Journal of Athletic Training (Allen Press). 2018;53[7]:687–95.

101. Ohji S, Aizawa J, Hirohata K, Ohmi T, Mitomo S, Jinno T, et al. Single-leg hop distance normalized to body height is associated with the return to sports after anterior cruciate ligament reconstruction. Journal of Experimental Orthopaedics. 2021;8[1]:26.

102. Palmieri-Smith RM, Lepley LK. Quadriceps strength asymmetry after anterior cruciate ligament reconstruction alters knee joint biomechanics and functional performance at time of return to activity. American journal of sports medicine. 2015;43[7]:1662–9.

103. Patterson B, Culvenor AG, Barton CJ, Guermazi A, Stefanik J, Morris HG, et al. Poor functional performance 1 year after ACL reconstruction increases the risk of early osteoarthritis progression. British journal of sports medicine. 2020;54[9]:546–53.

104. Peebles AT, Renner KE, Queen RM, Miller TK, Moskal JT. Associations between distance and loading symmetry during return to sport hop testing. Medicine & Science in Sports & Exercise. 2019;51[4]:624–9.

105. Piussi R, Beischer S, Thomeé R, Hamrin Senorski E. Superior knee self-efficacy and quality of life throughout the first year in patients who recover symmetrical muscle function after ACL reconstruction. Knee surgery, sports traumatology, arthroscopy. 2020;28[2]:555–67.

106. Pua YH, Ho JY, Chan SAS, Khoo SJ, Chong HC. Associations of isokinetic and isotonic knee strength with knee function and activity level after anterior cruciate

ligament reconstruction: a prospective cohort study. The Knee. 2017;24[5]:1067–74.

107. Raoul T, Klouche S, Lefevre N, Herman S, Guerrier B, El Hariri B, et al. Aptitude physique et psychologique pour la reprise du sport apres reconstruction du ligament croise anterieur du genou : score ACL-RSI et tests fonctionnels. Journal de traumatologie du sport. 2018;35[1]:15–24.

108. Raoul T, Klouche S, Guerrier B, El-Hariri B, Herman S, Gerometta A, et al. Are athletes able to resume sport at six-month mean follow-up after anterior cruciate ligament reconstruction? Prospective functional and psychological assessment from the French Anterior Cruciate Ligament Study (FAST) cohort. The Knee. 2019;26[1]:155–64.

109. Reinke EK, Spindler KP, Lorring D, Jones MH, Schmitz L, Flanigan DC, et al. Hop tests correlate with IKDC and KOOS at minimum of 2 years after primary ACL reconstruction. Knee surgery, sports traumatology, arthroscopy : official journal of the ESSKA. 2011;19[11]:1806–16.

110. Runer A, Suter A, di Sarsina TR, Jucho L, Gfoller P, Csapo R, et al. Quadriceps tendon autograft for primary anterior cruciate ligament reconstruction show comparable clinical, functional, and patient reported outcome measurements, but lower donor site morbidity compared with hamstring tendon autograft: A matched-pairs study with a mean follow-up of 6.5 years. Journal of ISAKOS : joint disorders & orthopaedic sports medicine. 2022;

111. Salatkaitė S, Šiupšinskas L, Žumbakytė-Šermukšnienė R, Gudas R. Eight-months after anterior cruciate ligament reconstruction: Is it time to return to physical activity? Baltic Journal of Sport & Health Sciences. 2021;121[2]:21–7.

112. San Jose AT, Maniar N, Timmins RG, Beerworth K, Hampel C, Tyson N, et al. Explosive hamstrings strength asymmetry persists despite maximal hamstring strength recovery following anterior cruciate ligament reconstruction using hamstring tendon autografts. Knee surgery, sports traumatology, arthroscopy : official journal of the ESSKA. 2023;31[1]:299–307.

113. Siney H, Bovard M, Del Gaudio N, Belmahfoud R, Boffa JF, Yahia N, et al. Muscle strength assessment after anterior cruciate ligament reconstruction: Is there a correlation between isokinetic test and one-legged hop test? Lettre de Medecine Physique et de Readaptation. 2010;26[1]:28–37.

114. Šipka N. Izidi funkcionalnih meritev in subjektivne ocene 6 mesecev po rekonstrukciji sprednje križne vezi s presadkom iz kit mišic fleksorjev kolena. / Functional and patient-self reported outcomes 6 months after anterior cruciate ligament reconstruction using hamstring tendons autograft. Fizioterapija. 2019;27[2]:Aug-15.

115. Stener S, Ejerhed L, Sernert N, Laxdal G, Rostgård-Christensen L, Kartus J. A long-term, prospective, randomized study comparing biodegradable and metal interference screws in anterior cruciate ligament reconstruction surgery: radiographic results and clinical outcome. American journal of sports medicine. 2010;38[8]:1598-1605.

116. Stropnik D, Sajovic M, Kacin A, Pavlic-Zaloznik S, Drobnic M. Early clinical and neuromuscular properties in patients with normal or sub-normal subjective knee function after anterior cruciate ligament reconstruction. Archives of orthopaedic and trauma surgery. 2020;140[9]:1231–9.

117. Thomeé R, Neeter C, Gustavsson A, Thomeé P, Augustsson J, Eriksson B, et al. Variability in leg muscle power and hop performance after anterior cruciate ligament reconstruction. Knee surgery, sports traumatology, arthroscopy. 2012;20[6]:1143–51.

118. Thompson XD, Bruce AS, Kaur M, Diduch DR, Brockmeier SF, Miller MD, et al. Disagreement in pass rates between strength and performance tests in patients recovering from anterior cruciate ligament reconstruction. The American journal of sports medicine. 2022;50[8]:2111–8.

119. Tourville TW, Jarrell KM, Naud S, Slauterbeck JR, Johnson RJ, Beynnon BD. Relationship between isokinetic strength and tibiofemoral joint space width changes after anterior cruciate ligament reconstruction. American journal of sports medicine. 2014;42[2]:302–11.

120. Tyler TF, Nicholas SJ, Hershman EB, Glace BW, Mullaney MJ, McHugh MP. The effect of creatine supplementation on strength recovery after anterior cruciate ligament (ACL) reconstruction: a randomized, placebo-controlled, double-blind trial. American journal of sports medicine. 2004;32[2]:383-388.

121. Ueda Y, Matsushita T, Shibata Y, Takiguchi K, Ono K, Kida A, et al. Association between meeting return-to-sport criteria and psychological readiness to return to sport after anterior cruciate ligament reconstruction. Orthopaedic Journal of Sports Medicine. 2022;10[5].

122. Ueda Y, Matsushita T, Shibata Y, Takiguchi K, Ono K, Kida A, et al. Satisfaction with playing pre-injury sports 1 year after anterior cruciate ligament reconstruction using a hamstring autograft. The Knee. 2021;33:282–9.

123. Ventura A, Iori S, Legnani C, Terzaghi C, Borgo E, Albisetti W. Single-bundle versus double-bundle anterior cruciate ligament reconstruction: assessment with vertical jump test. Arthroscopy. 2013;29[7]:1201-1210.

124. Vermesan D, Prejbeanu R, Laitin S, Georgianu V, Haragus H, Nitescu S, et al. Meniscal tears left in situ during anatomic single bundle anterior cruciate ligament

reconstruction. European review for medical and pharmacological sciences. 2014;18[2]:252–6.

125. Vijayan S, Cherukuri SM, Kulkarni MS, Naik MA, Shetty S, Bhat V, et al. Functional evaluation of anterior cruciate ligament reconstruction – comparison of two graft fixation techniques in the femoral tunnel. Muscles, Ligaments and Tendons Journal. 2021;11[1]:62–8.

126. Vijayan S, Kyalakond H, Kulkarni MS, Aroor MN, Shetty S, Bhat V, et al. Clinical outcome of anterior cruciate ligament reconstruction with modified transtibial and anteromedial portal. Musculoskeletal surgery. 2021;

127. Webster KE, Nagelli CV, Hewett TE, Feller JA. Factors associated with psychological readiness to return to sport after anterior cruciate ligament reconstruction surgery. American journal of sports medicine. 2018;46[7]:1545–50.

128. Webster K, Feller J, Webster KE, Feller JA. Younger patients and men achieve higher outcome scores than older patients and women after anterior cruciate ligament reconstruction. Clinical Orthopaedics & Related Research. 2017;475[10]:2472–80.

129. Welling W, Benjaminse A, Lemmink K, Gokeler A. Passing return to sports tests after ACL reconstruction is associated with greater likelihood for return to sport but fail to identify second injury risk. Knee. 2020;27[3]:949–57.

130. Welling W, Benjaminse A, Seil R, Lemmink K, Gokeler A. Altered movement during single leg hop test after ACL reconstruction: implications to incorporate 2-D video movement analysis for hop tests. Knee surgery, sports traumatology, arthroscopy. 2018;26[10]:3012–9.

131. Welling W, Benjaminse A, Seil R, Lemmink K, Zaffagnini S, Gokeler A. Low rates of patients meeting return to sport criteria 9 months after anterior cruciate ligament reconstruction: a prospective longitudinal study. Knee surgery, sports traumatology, arthroscopy. 2018;26[12]:3636–44.

132. Wellsandt E, Failla MJ, Axe MJ, Snyder-Mackler L. Does anterior cruciate ligament reconstruction improve functional and radiographic outcomes over nonoperative management 5 years after injury? American journal of sports medicine. 2018;46[9]:2103–12.

133. Wipfler B, Donner S, Zechmann CM, Springer J, Siebold R, Paessler HH. Anterior cruciate ligament reconstruction using patellar tendon versus hamstring tendon: a prospective comparative study with 9-year follow-up. Arthroscopy. 2011;27[5]:653-665.

134. Yuya U, Takehiko M, Yohei S, Kohei T, Akihiro K, Daisuke A, et al. Longitudinal quadriceps strength recovery after anterior cruciate ligament reconstruction with hamstring autograft: Patients stratified by preoperative quadriceps strength deficit. Journal of sport rehabilitation. 2020;29[5]:602–7.

135. Zhi-Cheng L, Yan L, Qi-Liang Z. Changes in the flexion and extension muscle strength and motor function of the knee joint after anterior cruciate ligament reconstruction. Chinese Journal of Tissue Engineering Research. 2018;22[27]:4393–9.

136. Zumstein F, Centner C, Ritzmann R. How limb dominance influences limb symmetry in ACL patients: effects on functional performance. BMC Sports Science, Medicine and Rehabilitation. 2022;14[1]:206.
